# Numerical convergence of the scalar auxiliary variable method applied to nonlinear stiff string models

**DOI:** 10.1007/s11071-026-12708-0

**Published:** 2026-06-29

**Authors:** Riccardo Russo, Michele Ducceschi, Stefan Bilbao

**Affiliations:** 1 Department of Industrial Engineering, University of Bologna, Viale Risorgimento 2, 40136 Bologna, Italy; 2 STMS (UMR9912), CNRS, IRCAM, Sorbonne Université, 1, place Igor Stravinsky, 75004 Paris, France

**Keywords:** Scalar auxiliary variable, Potential energy split, Numerical convergence, Nonlinear string, Geometric nonlinearities, Potential shift

## Abstract

This work is concerned with the Scalar Auxiliary Variable (SAV) method applied to geometrically nonlinear string models, focusing on the analysis of numerical convergence across various model formulations. An ODE system with a potential akin to that of the geometrically exact string is first analysed, providing both mathematical and numerical insight. Then, three nonlinear stiff string models are considered: geometrically exact (in two formulations), cubic, and Kirchhoff–Carrier models, each incorporating a stiffness term based on the Euler–Bernoulli formulation. A unified mathematical description is introduced in the continuous domain, using both quadratically split and non-split forms. Spatial and temporal discretisation is performed using Finite-Difference Time-Domain (FDTD) methods. Convergence is assessed by comparing SAV solutions with benchmark results obtained from convergent reference integrators at high sample rates, considering the transverse displacement, longitudinal displacement (when present), and the auxiliary variable. Results show that the non-split SAV converges for all three models, but only when the spatial step remains above the stability threshold of the corresponding split version, despite the scheme being unconditionally stable; furthermore, the global error is further strongly dependent on a potential shift constant. The split formulation converges reliably only when the potential decomposition reflects a natural separation between linear and nonlinear dynamics, as in the cubic and Kirchhoff–Carrier models, and in one particular form of the geometrically exact string.

## Introduction

When a string undergoes moderate amplitude vibration, Hooke’s law is typically assumed valid [1, 2], with nonlinearities arising solely from the geometry. Early studies on nonlinear strings can be traced back to Kirchhoff [3] and Carrier [4], followed by Anand [5] and Murthy and Ramakrishna [6], who formalised the Kirchhoff–Carrier string [7]. This model primarily reproduces tension modulation [8], and spectral enrichment under finite-impedance boundary conditions [9].

In the late 1960 s, Narasimha [10], Anand [5], and Morse [1] proposed a three-dimensional string model commonly referred to as geometrically exact [11], capturing pointwise coupling between transverse and longitudinal motion. Longitudinal modes induce nonlinear mixing, generating mode coupling and phantom partials [2, 12]. Simpler models are usually obtained by expanding the geometrically exact potential in Taylor series. Anand’s form retains terms up to third order and yields a positive-definite Hamiltonian [13]. Further simplifications produce the cubic model (neglecting longitudinal motion) [13] or the Kirchhoff–Carrier equation (neglecting longitudinal inertia) [14].

These string models do not admit closed-form solutions in the general case [15], and numerical methods are therefore required. A major concern is ensuring numerical stability, particularly in acoustics, where energy dissipation is typically low. A common approach is to enforce the conservation of a discrete quantity with the dimensions of energy (henceforth *energy* or *numerical energy*), in such a way that the norm of the solution remains bounded over time [16]. Relevant examples in the context of string modelling include the work of Bilbao [7, 13, 17] and Chabassier [11, 18, 19]. Aside from the Kirchhoff-Carrier model, which admits an explicit solver, the energy method typically leads to either linearly implicit or fully implicit schemes. Beyond the computational bottleneck introduced in either case, fully implicit schemes incur variable per-time-step costs and require selecting iteration caps and tolerance thresholds [20]. Furthermore, the existence and uniqueness of the numerical solution are not always guaranteed [21].

Three energy-conserving numerical methods have been developed lately, based on potential quadratisation. Lopes, Hélie, and Falaize introduced a port-Hamiltonian approach [22, 23], which retains the number of variables for invertible potentials and univariate scalar cases. The Invariant Energy Quadratisation (IEQ) method [24–26] introduces a single additional variable and handles multivariate, non-invertible potentials. The Scalar Auxiliary Variable (SAV) method [27–29] extends IEQ by treating the nonlinear energy as a scalar, enabling fully explicit solvers [30, 31] when using the Sherman–Morrison formula [32]. SAV has been applied to nonlinear strings using Finite Difference Time Domain (FDTD) methods for the Kirchhoff–Carrier and cubic models, as well as modal methods for the cubic model [33–35], while both FDTD [30, 31, 36] and the Finite Element Method (FEM) [37, 38] have been applied to the geometrically exact string. SAV with FEM has also been employed to simulate geometrically nonlinear structures in a non-canonical Hamiltonian formulation [39]. A common approach is to split the potential into quadratic and residual parts, with SAV applied to the latter [31].

SAV has been shown to outperform implicit methods in terms of efficiency. However, although its stability and consistency properties have been verified both mathematically and numerically [34, 38], convergence remains an open question. A formal proof exists for diffusion-type nonlinearities [28, 38], but this does not extend to gradient-dependent potentials (i.e. wave equations). Numerical pollution has been observed in various contexts [33, 34, 38, 40], which has motivated the development of regularisation techniques [33, 34, 41, 42] and investigations into the role of the potential shift [43]. Numerical approaches have demonstrated convergence in the case of collisions [41]. The same methodology has been applied to geometric nonlinearities, for the Kirchhoff–Carrier and cubic string models using FDTD [33, 34], and for the geometrically exact string using FEM [38]. In the latter work, Castera observed convergence only for specific space–time step pairs, typically obeying the Courant-Friedrichs-Lewy (CFL) limit [44], even in unconditionally stable cases. The best performance was observed when the quadratic potential was treated with SAV, contrary to what was proposed in [31]. Distinctions were also noted between equivalent formulations of the geometrically exact string. None of these studies verified convergence of the auxiliary variable.

The present study aims to conduct a systematic numerical convergence analysis of SAV applied to three nonlinear, stiff string models: geometrically exact (with three different forms of the potential), cubic, and models of Kirchhoff–Carrier type. First, SAV is analysed for a system of ordinary differential equations (ODEs) with a potential akin to the geometrically exact, examining global error behaviour both mathematically and numerically. A numerical study then follows for the string models. Each includes a stiffness term as per Euler-Bernoulli [45], and is restricted to planar vibration, as is common in many studies [7]. Spatial discretisation is based on FDTD methods, and all strings share the same physical parameters and initial conditions. Convergence is monitored for the transverse displacement, longitudinal displacement (when present), and the auxiliary variable, and SAV is studied both in full form (by including all the potential into the SAV formulation) and in split form (by treating the quadratic part with classic techniques).

The remainder of this work is structured as follows. Sect. 2 introduces the Cauchy problem in the continuous domain, outlines ODE potentials, and develops potential quadratisation for ODEs. Sect. 3 covers time discretisation and derives the SAV algorithms. Sect. 4 reports the ODE test results. Sect. 5 establishes a unified mathematical formulation for string models in the continuous domain, describes the different string potentials, and extends potential quadratisation to PDEs. Sect. 6 addresses spatial discretisation, while Sect. 7 derives SAV for PDEs. Sect. 8 reports the numerical testing procedure and results. Finally, Sects. 9 and 10 summarise the main findings and draw conclusions.

## ODE system

Consider a set of two time-dependent ODEs written in terms of the vector variable ${{\textbf{w}}} = {{\textbf{w}}}(t) = [u(t),v(t)]^\intercal : \mathbb {R}^+_0 \rightarrow \mathbb {R}^2$, associated with a two-degree-of-freedom system. The system evolves according to the Euler-Lagrange equation:

1 $$\begin{aligned} \frac{\textrm{d}}{\textrm{d t}} \left( \nabla _{{\dot{\textbf{w}}}} K \right) = -\nabla _\textbf{w}V, \end{aligned}$$

where $K = \tfrac{1}{2}{{\dot{\textbf{w}}}}^\intercal {{\dot{\textbf{w}}}}$ denotes the kinetic energy, and $V = V(u,v): \mathbb {R}^2 \rightarrow \mathbb {R}^+_0$ is a potential function assumed bounded from below, the explicit form of which will be specified later.

All potential functions considered here can be written as the sum of a non-negative quadratic contribution *Q* and a residual term *R*:

2 $$\begin{aligned} V = Q + R. \end{aligned}$$

Such a decomposition is commonly exploited in numerical methods, allowing the quadratic and residual parts to be handled by distinct techniques [31]. A Cauchy problem is obtained by specifying initial conditions:

3 $$\begin{aligned} \textbf{w}(0) = \textbf{w}_0, \qquad {{\dot{\textbf{w}}}}(0) = {{\dot{\textbf{w}}}}_0, \end{aligned}$$

fully determining the system’s time evolution.

### Potential quadratisation

The scalar auxiliary variable (SAV) reformulation is now written in a unified form, yielding both a *non-split* (NS) and a *split* (S) variant, as follows. In the split setting, the potential is decomposed as per (2), with quadratic contribution:

4 $$\begin{aligned} Q(\textbf{w}) \triangleq \tfrac{1}{2}\, \textbf{w}^\intercal \textbf{K}\,\textbf{w}\ge 0; \end{aligned}$$

so that:

5 $$\begin{aligned} \nabla _\textbf{w}Q = \textbf{K}\textbf{w}, \end{aligned}$$

where $\textbf{K}$ is symmetric positive definite. In the non-split case, no decomposition is introduced.

Both variants are expressed compactly by introducing a binary switch $\sigma \in \{0,1\}$ and defining the target potential:

6 $$\begin{aligned} \Phi \triangleq \sigma \, V + (1-\sigma )\, R. \end{aligned}$$

The choice $\sigma =1$ corresponds to NS, while $\sigma =0$ corresponds to S. Assuming $\Phi \ge 0$, define the auxiliary variable:

7 $$\begin{aligned} \psi \triangleq \sqrt{2\Phi + \varepsilon }, \end{aligned}$$

with $\varepsilon >0$ being a regularisation constant, whose role in the convergence properties of the associated numerical schemes will be further explained below. By the chain rule:

8 $$\begin{aligned} {{\dot{\psi }}} = \left( \nabla _\textbf{w}\psi \right) ^\intercal \, {{\dot{\textbf{w}}}}. \end{aligned}$$

Differentiation of (7) yields:

9 $$\begin{aligned} \textbf{g}\triangleq \nabla _\textbf{w}\psi = \frac{\nabla _\textbf{w}\Phi }{\sqrt{2\Phi +\varepsilon }}; \end{aligned}$$

so that:

10 $$\begin{aligned} \nabla _\textbf{w}\Phi = \psi \,\textbf{g}. \end{aligned}$$

Using (10), the SAV reformulation becomes [31]:

11a $$\begin{aligned} \ddot{\textbf{w}}&= -(1-\sigma )\,\textbf{K}\textbf{w}- \psi \,\textbf{g}, \end{aligned}$$

11b $$\begin{aligned} {{\dot{\psi }}}&= \textbf{g}^\intercal {{\dot{\textbf{w}}}}. \end{aligned}$$

In addition to (3), a new initial condition is required:

$$ \psi (0) = \sqrt{2\Phi (\textbf{w}_0)+\varepsilon }. $$

Left-multiplying (11a) by ${{\dot{\textbf{w}}}}^\intercal$ and using (11b) yields the continuous energy balance:

12 $$\begin{aligned} \dot{H} = 0 \implies H(t) = H(0) \triangleq H_0, \end{aligned}$$

where the total energy is:

13 $$\begin{aligned} H = K + (1-\sigma )\,Q + \frac{\psi ^2}{2}, \end{aligned}$$

which is non-negative.

### ODE potentials

A simplified potential function that resembles the geometrically exact potential for tensioned strings may be expressed as:

14a $$\begin{aligned} Q_\textrm{A}&= \frac{ u^2}{2} + \dfrac{v^2}{2} \end{aligned}$$

14b $$\begin{aligned} R_\textrm{A}&= \dfrac{ \alpha -1}{2}\left( \sqrt{(1+v)^2+u^2}-1 \right) ^2, \end{aligned}$$

with $V \triangleq Q_\textrm{A} + R_\textrm{A}$. Boundedness from below of *V* is evident as both components in (14) are non-negative under the assumption, assumed valid, that $\alpha \ge 1$.

**Fig. 1:**
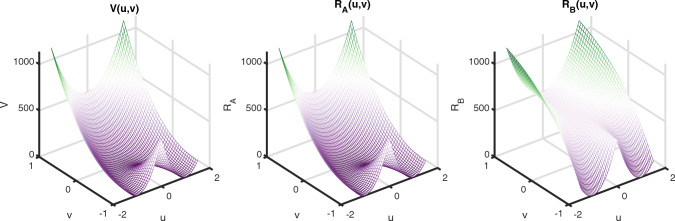
SAV potentials considered in the ODE study for $\alpha = 10000$

For small *u*, the potential reduces to:

15 $$\begin{aligned} V \approx \frac{u^2}{2} + \frac{\alpha v^2}{2}, \end{aligned}$$

so that the dynamics consist of two uncoupled simple harmonic oscillators:

$$ \ddot{u} \approx -u, \qquad \ddot{v} \approx -\alpha v. $$

This observation motivates an alternative decomposition of the geometrically exact potential, distinct from (14), in which the quadratic part reproduces the linearised oscillator energy, while the remaining nonlinear contribution is treated separately. Consider therefore the splitting:

16a $$\begin{aligned} Q_\textrm{B}&= \frac{u^2}{2} + \frac{\alpha v^2}{2}, \end{aligned}$$

16b $$\begin{aligned} R_\textrm{B}&= (\alpha - 1) \left( \frac{u^2}{2} + \frac{3}{2} + v - \sqrt{(1+v)^2 + u^2} \right) , \end{aligned}$$

where the quadratic component $Q_\textrm{B}$ coincides exactly with the oscillator potential (15). In this case, one has $V = Q_\textrm{B} + R_\textrm{B} - \tfrac{\alpha - 1}{2}$: the added constant is necessary for the residual to be non-negative, but does not affect the motion. In [38], a splitting analogous to (48) is proposed; however, the residual is shifted by a different constant, so that it changes sign over parts of the admissible domain and thus violates the non-negativity requirement underlying quadratisation.

Under the assumption $v > -1$, the triangle inequality:

$$ \sqrt{(1+v)^2 + u^2} \le (1+v) + |u|, $$

implies $R_\textrm{B} \ge 0$, so that the residual contribution is non-negative. Note that, in the spatially distributed case, this constraint ensures that the string does not fold back on itself, which would be unphysical [46]. Indeed, while this constraint cannot be guaranteed to hold a priori, any violation of $v > -1$ corresponds to a loss of orientation-preservation of the deformation map, and thus to a regime that lies outside the domain of validity of the geometrically exact model itself. System $R_\textrm{B}$ is included in the analysis as it is the only splitting that allows for separating the linear and nonlinear parts of the potential energy entirely.

The three potentials are shown in the admissible domain $\{(u,v):\, v>-1\}$ in Fig. 1.

#### Well-posedness

Formal statements and proofs are deferred to Appendix A, so as not to interrupt the main development. Only the structure of the estimates relevant to the present discussion is summarised here.

System (11) is written as a first-order system for ${{\textbf{x}}} = (\textbf{w},{{\dot{\textbf{w}}}},\psi )^\intercal$. Local well-posedness follows from the Picard–Lindelöf theorem once the right-hand side is locally Lipschitz. The linear contribution $(1-\sigma ){\textbf{K}} \textbf{w}$ is globally Lipschitz with constant $(1-\sigma )\lambda _{\max }({\textbf{K}})$, while the nonlinear part is governed by $\psi \,\textbf{g}$.

Let $G_0$ denote the supremum of $\textbf{g}$ on a bounded set and let $\mathcal {C}_g$ denote its Lipschitz constant. A Lipschitz constant for the full right-hand side can then be written in the form (see Appendix A):

17 $$\begin{aligned} \begin{aligned} \mathcal {C} = \max \bigl (&(1\!-\!\sigma )\,\lambda _{\max }(\textbf{K}) \\&+ 2\sqrt{2 H_0}\,\mathcal {C}_g, \;\;1 + G_0 \bigr ), \end{aligned} \end{aligned}$$

which depends linearly on the eigenvalues of ${\textbf{K}}$ through the factor $(1-\sigma )$. In particular, for $\sigma =1$ (NS) the linear contribution vanishes, while for $\sigma =0$ (S) it is fully retained. For the specific split form B considered below, the residual structure ensures that no additional restriction arises from the quadratic component.

Since $\psi = \sqrt{2\Phi +\varepsilon }$ satisfies $\psi \ge \sqrt{\varepsilon }$, positivity of $\varepsilon$ guarantees regularity of the vector field and therefore local existence and uniqueness for admissible initial data. However, $\mathcal {C}_g$ may depend explicitly on $\varepsilon$. As $\varepsilon \rightarrow 0$, the Lipschitz constant may grow, directly affecting stability margins and convergence rates of the time-discrete schemes.

Thus, although the continuous problem is well posed for any fixed $\varepsilon >0$, the behaviour of $\textbf{g}$, in particular its size and Lipschitz constant, plays a decisive role. A detailed analysis of these quantities is postponed to Appendix A, but it is precisely this $\varepsilon$-dependence that underpins the convergence behaviour investigated in the following sections.

## Time discretisation

Approximate solutions to the Cauchy problems are obtained after sampling time with a small step size $k \in \mathbb {R}^+$, corresponding to a sampling frequency $f_s = 1/k$. A state vector $\textbf{w}(t)$ is thus approximated at discrete times $t = nk, \; n \in \mathbb {N}$ by the sequence $\textbf{w}^n \approx \textbf{w}(nk)$. Time difference operators can then be introduced as operators acting on the time series:

18a $$\begin{aligned}&\delta _{t\pm } \textbf{w}^n= \pm \frac{\textbf{w}^{n\pm 1} - \textbf{w}^n}{k}, \end{aligned}$$

18b $$\begin{aligned}&\delta _{t\cdot }\textbf{w}^n= \frac{\textbf{w}^{n+1}- \textbf{w}^{n-1}}{2k}. \end{aligned}$$

The first corresponds to forward and backward one-step difference operators, while the latter represents a centred difference operator. A discrete approximation of the second derivative follows from their composition:

19 $$\begin{aligned} \delta _{tt}\textbf{u}^n= \delta _{ t+}\delta _{t-}\textbf{u}= \frac{\textbf{u}^{n+1}- 2\textbf{u}^n+ \textbf{u}^{n-1}}{k^2}. \end{aligned}$$

In a similar way, time-averaging operators are defined as:

20 $$\begin{aligned} \mu _{t\pm } \textbf{u}^n= \frac{\textbf{u}^{n\pm 1} + \textbf{u}^n}{2}. \end{aligned}$$

These constructions also extend to sequences defined on staggered time grids. For example, for the quantity $\psi ^{n-\frac{1}{2}} \approx \psi ((n-\frac{1}{2})k)$, one has: $\delta _{ t+}\psi ^{n-\frac{1}{2}} = (\psi ^{n+\frac{1}{2}} - \psi ^{n-\frac{1}{2}})/k$. One-step operators acting on the sequences $\textbf{u}$ and $\textbf{v}$ are first-order accurate, whereas the centred operators achieve second-order accuracy. When applied over staggered grids, one-step operators are also second-order accurate. For further details on the accuracy of these operators, see [34].

### SAV time-stepping scheme

A single time-discrete formulation covering both the non-split (NS) and split (S) cases is obtained directly from (11) by retaining the switch $\sigma \in \{0,1\}$. Following [31], consider:

21a $$\begin{aligned}&\delta _{tt}\textbf{w}^n = -(1-\sigma )\,\textbf{K}\textbf{w}^n - \mu _{ t+}\psi ^{n-\frac{1}{2}} \textbf{g}^n \end{aligned}$$

21b $$\begin{aligned}&\delta _{ t+}\psi ^{n-\frac{1}{2}} = (\textbf{g}^n)^\intercal \delta _{t\cdot }\textbf{w}^n, \end{aligned}$$

with $\textbf{g}^n = \textbf{g}(\textbf{w}^n)$. The auxiliary variable $\psi ^{n-\frac{1}{2}}$ is treated as a staggered time series and updated via the discrete chain rule (21b).

Expanding the difference operators in (21a) yields the update:

22 $$\begin{aligned} \textbf{A}^n \textbf{w}^{n+1} =\textbf{B}\textbf{w}^n + \textbf{C}^n\textbf{w}^{n-1} - k^2 \textbf{g}^n \psi ^{n-\frac{1}{2}}. \end{aligned}$$

The matrices take the form:

23a $$\begin{aligned} \textbf{A}^n&\triangleq \textbf{I} +\frac{k^2}{4} \textbf{g}^n(\textbf{g}^n)^\intercal \end{aligned}$$

23b $$\begin{aligned} \textbf{B}&\triangleq 2\textbf{I} +k^2(1-\sigma )\textbf{K},\end{aligned}$$

23c $$\begin{aligned} \textbf{C}^n&\triangleq -\textbf{I} +\frac{k^2}{4} \textbf{g}^n(\textbf{g}^n)^\intercal \end{aligned}$$

Left-multiplying (21a) by $\left( \delta _{t\cdot }\textbf{w}^n \right) ^\intercal$ and using (21b) yields the discrete energy balance:

24 $$\begin{aligned} \delta _{ t+}\mathfrak {h}^{n-\frac{1}{2}} = 0, \end{aligned}$$

with discrete energy:

25 $$\begin{aligned} \mathfrak {h}^{n-\frac{1}{2}} = \mathfrak {K}^{n-\frac{1}{2}} + (1-\sigma )\, \mathfrak {Q}^{n-\frac{1}{2}} + \frac{\left( \psi ^{n-\frac{1}{2}} \right) ^2}{2}. \end{aligned}$$

Here $\mathfrak {K}^{n-\frac{1}{2}}$ denotes the discrete kinetic energy and $\mathfrak {Q}^{n-\frac{1}{2}}$ the quadratic contribution associated with $\textbf{K}$. Non-negativity of the quadratic contribution requires the condition [7]:

26 $$\begin{aligned} 4 - k^2 (1-\sigma )\, \lambda _{\max }(\textbf{K}) > 0. \end{aligned}$$

For $\sigma =1$ (NS), this constraint vanishes, and the scheme is unconditionally stable. For $\sigma =0$ (S), (26) reduces to the standard CFL-type condition involving $\lambda _{\max }(\textbf{K})$. Under (26), all energy contributions are non-negative, and the method is energy-conservative and stable.

### Numerical behaviour of the SAV schemes

Convergence results for the SAV discretisations are given in Appendix B. On any finite time interval, the schemes are globally convergent and second-order accurate, under the stability condition (26) and provided that ${{\textbf{g}}}$ is Lipschitz-continuous on the set visited by the discrete solution. Lipschitz continuity of ${{\textbf{g}}}$ follows from boundedness of its Jacobian ${\textbf{J}} \textbf{g}$. The behaviour of ${\textbf{J}} \textbf{g}$ in a neighbourhood of equilibrium therefore determines the constants appearing in the global error estimates.

For scheme NS, and for scheme S based on the residual $R_\textrm{A}$, the associated SAV potential vanishes quadratically at (0, 0). Locally, one has:

$$ \Phi (\textbf{w}) = \mathcal {O}(\Vert \textbf{w}\Vert ^2), \qquad \psi = \sqrt{2\Phi +\varepsilon }. $$

In this case, the regularisation parameter $\varepsilon$ enters directly into the scaling of the Jacobian, and one obtains:

$$ \Vert {\textbf{J}} \textbf{g}\Vert = \mathcal {O}(\varepsilon ^{-1/2}) \quad \text {at } (0,0). $$

Thus, although any fixed $\varepsilon >0$ ensures well-posedness, the Lipschitz constant of ${{\textbf{g}}}$ grows as $\varepsilon \rightarrow 0^+$. The corresponding error bounds deteriorate accordingly.

For the shifted residual $R_\textrm{B}$, the situation differs. Here:

$$ R_\textrm{B}(0,0) = \frac{\alpha -1}{2} > 0, $$

so the SAV potential does not vanish at the equilibrium. Consequently, $\psi$ remains uniformly bounded away from zero, independently of $\varepsilon$, and ${\textbf{J}} \textbf{g}$ remains bounded as $\varepsilon \rightarrow 0^+$. The Lipschitz constant of ${{\textbf{g}}}$ is therefore insensitive to $\varepsilon$ in this case, and the associated convergence behaviour is correspondingly more robust. The distinction arises solely from whether the SAV potential vanishes at equilibrium, and not from the order of the underlying time discretisation.

## Numerical tests

As a numerical test, reference solutions are computed via the Stormer-Verlet algorithm run with a very small time step. Then, SAV solutions are computed at progressively smaller time steps, and the error with respect to the benchmark solutions is evaluated at a pre-defined, fixed time instant $t_E$ as:

27 $$\begin{aligned} E(t_E) = |{\hat{u}}^{{\hat{n}}_E} - u^{n_E}|, \end{aligned}$$

where ${\hat{u}}^{{\hat{n}}_E}$ denotes the reference solution computed at ${\hat{n}}_E \triangleq t_E/{\hat{k}}$, with ${\hat{k}}$ representing the time step of the reference solution. This procedure is carried out for ${u,v,\psi }$, and the error for $\psi$ is evaluated at a staggered time instant $t_E - \tfrac{k}{2}$. No results are reported here regarding the conservation of numerical energy or the consistency of the SAV algorithms, as these aspects have been extensively addressed in previous publications (see Sect. 1 for a thorough list).

**Fig. 2:**
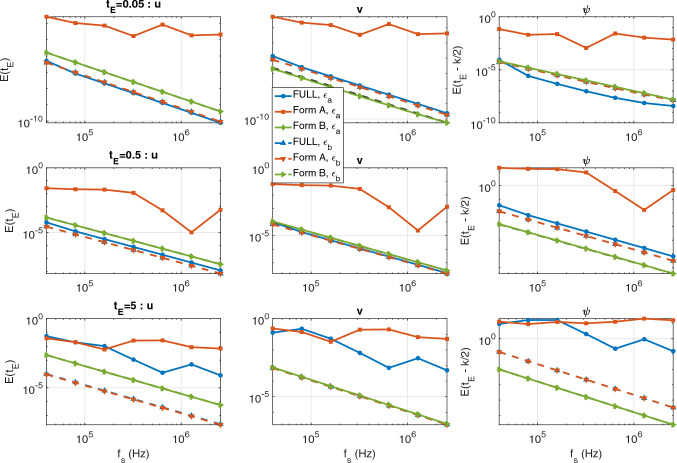
Convergence curves for the ODE tests, at $t_E = \{0.05, 0.5, 5\}$ s, and with $\varepsilon _a$ and $\varepsilon _b.$

A base audio sampling rate of 10000 Hz is constantly used, which is then increased with an integer oversampling factor (OF), such that the final rate is: $f_s = \text {OF} \times 10000$. The oversampling value is always expressed as a power of 2, and thus OF $= 2^a$, with $a\in \mathbb {N}$. The SAV schemes are run with $a \in [1,8]$, while the benchmark integrator is obtained with $a = 10$. The physical constant is set to $\alpha = 10^5$, and systems are initialised with $\textbf{w}_0 = [0.5,0.2]^\intercal$ and ${{\dot{\textbf{w}}}} = \textbf{0}$. Tests are repeated for $\varepsilon = 2.2204\times 10^{-16} \triangleq \varepsilon _a$ (corresponding to machine epsilon) and $\varepsilon = 10^3 \triangleq \varepsilon _b$, and for three values of $t_E$. Results are reported in Fig. 2. Schemes *V* and $R_\textrm{A}$ exhibit poor convergence properties, especially at longer simulation times, whereas using $\varepsilon = 1000$ stabilises the convergence curves. On the other hand, scheme $R_\textrm{B}$’s convergence properties are not affected by the regularisation constant. These results confirm the theoretical predictions of Appendix B.

## Continuous string models

This section introduces the three models of string vibration in the continuous domain, ordered by decreasing complexity. Using a variational formulation, a common form is given here as:

28 $$\begin{aligned} \partial _t \left( \triangledown _{\partial _t\boldsymbol{\textit{w}}} \textrm{K} \right) = -\triangledown _{\boldsymbol{\textit{w}}} \textrm{V}, \end{aligned}$$

expressed via the kinetic and potential energy functionals $\textrm{K},\textrm{V}$ to be defined shortly, and their associated variational gradients expressed via the symbol $\triangledown$, also illustrated below. The vector $\boldsymbol{\textit{w}}= [u,v]^\intercal$ denotes the string’s state, where the components $u = u(x,t)$, $v = v(x,t)$, with $u,v: [0,L] \times \mathbb {R}^+_0 \rightarrow \mathbb {R}$, represent the transverse and longitudinal displacements, respectively. The physical parameters are as follows: the length *L* of the unstretched string (m); the material density $\rho$ (kg $\hbox {m}^{-3}$); and the string’s cross-sectional area *A* ($\hbox {m}^2$). In what follows, $\partial ^i_j$ denotes the $i^{\text {th}}$ partial derivative with respect to coordinate *j*.

Assuming a constant density and cross-sectional area, the total kinetic energy is given by the functional $\textrm{K}$, with associated kinetic energy density $\mathcal {K}$:

29 $$\begin{aligned} \textrm{K} = \int _0^L {\mathcal {K}}\,\textrm{d}x; \quad {\mathcal {K}}= \frac{\rho A}{2} \partial _t {\boldsymbol{\textit{w}}}^\intercal \partial _t {\boldsymbol{\textit{w}}}\,. \end{aligned}$$

Given a generic functional $\textrm{J}$ of a function *f*(*x*), with $f: [a,b] \rightarrow \mathbb {R}$, defined as $\textrm{J}(f) = \int _a^b J(f(x),f'(x), f''(x))\,\textrm{d}x$, its functional (variational) derivative is defined via the first variation of the functional with respect to admissible test functions, assumed to belong to $C^2([a,b])$ and to satisfy homogeneous boundary conditions, so that boundary terms vanish. The resulting functional derivative is given explicitly as:

30 $$\begin{aligned} \frac{\delta \textrm{J}}{\delta f} = \frac{\partial J}{\partial f} - \frac{\textrm{d}}{\textrm{d}x} \left( \frac{\partial J}{\partial f'} \right) + \frac{\textrm{d}^2}{\textrm{d}x^2} \left( \frac{\partial J}{\partial f''} \right) . \end{aligned}$$

Applying this definition to $\textrm{K}$, the Euler-Lagrange inertial term on the left-hand side of (28) evaluates to:

31 $$\begin{aligned} \partial _t \left( \triangledown _{\partial _t\boldsymbol{\textit{w}}}\textrm{K}\right) \triangleq \partial _t \begin{bmatrix} \frac{\delta \textrm{K}}{\delta (\partial _t u)} \\ \frac{\delta \textrm{K}}{\delta (\partial _t v)} \end{bmatrix} = \rho A \partial _t^2 \boldsymbol{\textit{w}}, \end{aligned}$$

where $\delta /\delta (\partial _t u)$ and $\delta /\delta (\partial _t v)$ represent the functional derivatives with respect to the velocities. The total potential $\textrm{V}$ is a functional that depends on the specific string model. It is defined as:

32 $$\begin{aligned} \textrm{V} = \int _0^L \mathcal {V} (\zeta , \chi , \xi ) \,\textrm{d}x, \end{aligned}$$

where $\mathcal {V}$ is the associated potential density. For notational compactness, the following definitions are used: $\zeta \triangleq \partial _x u$, the string slope; $\chi \triangleq \partial _x^2 u$, the approximate curvature (proportional to the cross-section bending moment for relatively thin strings); and $\xi \triangleq \partial _x v$, the horizontal strain. In the Kirchhoff-Carrier and cubic models, the dependence of $\mathcal {V}$ is restricted to $\zeta$ and $\chi$, and the state vector reduces to its transverse component *u*.

Using (30), the variational gradient of $\textrm{V}$ results:

33 $$\begin{aligned} \triangledown _{\boldsymbol{\textit{w}}} \textrm{V} \triangleq \begin{bmatrix} \frac{\delta \textrm{V}}{\delta u} \\ \frac{\delta \textrm{V}}{\delta v} \end{bmatrix} = \begin{bmatrix}- \partial _x \partial _\zeta \mathcal {V} + \partial _x^2 \partial _\chi \mathcal {V}\\ -\partial _x \partial _\xi \mathcal {V}\end{bmatrix}, \end{aligned}$$

where $\delta /\delta u$ and $\delta /\delta v$ indicate the functional derivatives with respect to *u* and *v*, respectively.

Analogously to the ODE case treated in Sect. 2, all string potentials considered here may be expressed as a sum of a non-negative quadratic term $\mathcal {Q}$ and a residual contribution $\mathcal {R}$, such that:

34 $$\begin{aligned} \mathcal {V} = \mathcal {Q} + \mathcal {R}. \end{aligned}$$

An energy balance for system (28) is obtained by taking the $L^2$ inner product of the equation with the velocity $\partial _t \boldsymbol{\textit{w}}^\intercal$. Consider for simplicity the set of simply supported boundary conditions:

35 $$\begin{aligned} u\big |_{x=0,L} = \chi \big |_{x=0,L} = v\big |_{x=0,L} = 0 \quad \forall \, t \ge 0. \end{aligned}$$

By applying integration by parts along with the chain rule,

36 $$\begin{aligned} \partial _t \mathcal {V} = \partial _t \zeta \partial _\zeta \mathcal {V} + \partial _t \chi \partial _\chi \mathcal {V} + \partial _t \xi \partial _\xi \mathcal {V}, \end{aligned}$$

as well as Leibniz’s integration rule (assuming the potential density is sufficiently regular), one obtains the energy balance:

37 $$\begin{aligned} \dot{\textrm{H}} = 0; \quad \textrm{H} = \textrm{K} +\textrm{V}. \end{aligned}$$

The system is conservative, and the energy $\textrm{H}$ remains non-negative provided that the potential is itself non-negative.

### Potential quadratisation

The distributed formulation is written in unified form, including the regularising shift. Let:

$$ \mathrm V = \mathrm Q + \mathrm R, $$

with $\mathrm Q$ quadratic and $\mathrm R$ the remaining contribution, and introduce a switch $\sigma \in \{0,1\}$. Define the target functional:

38 $$\begin{aligned} \Phi \triangleq \sigma \,\mathrm V + (1-\sigma )\,\mathrm R, \end{aligned}$$

and assume $\Phi \ge 0$ on the admissible set. The scalar auxiliary variable is defined with shift $\varepsilon >0$ as:

39 $$\begin{aligned} \Psi \triangleq \sqrt{2\Phi + \varepsilon }. \end{aligned}$$

Using the variational gradient $\triangledown$, the chain rule gives:

40 $$\begin{aligned} \partial _t \Psi = \int _0^L \bigl ( \triangledown _{\boldsymbol{\textit{w}}}\Psi \bigr )^\intercal \partial _t \boldsymbol{\textit{w}}\,\mathrm d x. \end{aligned}$$

Define:

41 $$\begin{aligned} \boldsymbol{\textit{g}}\triangleq \triangledown _{\boldsymbol{\textit{w}}}\Psi = \frac{\triangledown _{\boldsymbol{\textit{w}}}\Phi }{\sqrt{2\Phi +\varepsilon }}, \end{aligned}$$

so that

42 $$\begin{aligned} \triangledown _{\boldsymbol{\textit{w}}}\Phi = \Psi \,\boldsymbol{\textit{g}}. \end{aligned}$$

The original equation (28) becomes:

43 $$\begin{aligned} \rho A \partial _t^2 \boldsymbol{\textit{w}}= -\triangledown _{\boldsymbol{\textit{w}}}\mathrm V = -(1-\sigma )\,\triangledown _{\boldsymbol{\textit{w}}}\mathrm Q - \Psi \,\boldsymbol{\textit{g}}. \end{aligned}$$

For the quadratic component:

44 $$\begin{aligned} \triangledown _{\boldsymbol{\textit{w}}}\mathrm Q = \mathcal {L} \boldsymbol{\textit{w}}, \end{aligned}$$

with $\mathcal {L}$ a linear, self-adjoint differential operator. The unified quadratised system therefore reads:

45a $$\begin{aligned} \rho A \partial _t^2 \boldsymbol{\textit{w}}&= -(1-\sigma )\,\mathcal {L} \boldsymbol{\textit{w}}- \Psi \,\boldsymbol{\textit{g}}, \end{aligned}$$

45b $$\begin{aligned} \partial _t \Psi&= \int _0^L \boldsymbol{\textit{g}}^\intercal \partial _t \boldsymbol{\textit{w}}\,\mathrm d x. \end{aligned}$$

For $\sigma =1$, the full potential is incorporated into $\Psi$. For $\sigma =0$, only the residual is quadratised and $\mathcal {L}\boldsymbol{\textit{w}}$ appears explicitly.

The energy balance remains that of (37), and the Hamiltonian can be written as:

46 $$\begin{aligned} \mathrm H = \mathrm K + (1-\sigma )\,\mathrm Q + \frac{\Psi ^2}{2}, \end{aligned}$$

which is non-negative.

### Geometrically exact model (G)

As mentioned in Sect. 1, the most complete model for string vibration (neglecting material nonlinearities) is the geometrically exact. This model accounts for pointwise coupling between transverse and longitudinal motion and can be expressed, in continuous time and space, in different equivalent forms. The original one, appearing in classical references such as the book by Morse and Ingard [1], does not possess a positive semi-definite nonlinear residual, and, as such, breaks the energy positivity property. An alternative form was proposed by Ducceschi and Bilbao [31, 36, 47]. Its potential density can be decomposed into two components, mirroring the ODE case (14), except for the addition of a linear stiffness term:

47a $$\begin{aligned} \mathcal {Q}_{\mathrm {G_A}}&= \frac{T_0}{2} \left( \zeta ^2 + \xi ^2 \right) + \frac{EI}{2}\chi ^2,\end{aligned}$$

47b $$\begin{aligned} \mathcal {R}_{\mathrm {G_A}}&= \frac{EA - T_0}{2} \left( \sqrt{\left( 1+\xi \right) ^2 + \zeta ^2} -1 \right) ^2. \end{aligned}$$

The full geometrically exact potential is written $\mathcal {V}_\textrm{G} \triangleq \mathcal {Q}_\mathrm {G_A} + \mathcal {R}_\mathrm {G_A}$. Both components are non-negative when $EA - T_0\ge 0$. The additional physical constants are defined as follows: $T_{0}$ is the tension at rest (N), *E* is Young’s modulus (Pa), and *I* is the area moment of inertia. For a string with circular cross-section, $I = \pi r^4/4$, where *r* is the radius in m.

A potential form that mirrors the ODE components (16), is:

48a $$\begin{aligned} \mathcal {Q}_{\mathrm {G_B}}&= \frac{T_0}{2} \zeta ^2 + \frac{EA}{2} \xi ^2 + \frac{EI}{2}\chi ^2,\end{aligned}$$

48b $$\begin{aligned} \mathcal {R}_{\mathrm {G_B}}&= (EA - T_0) \left( \frac{\zeta ^2}{2} + \frac{3}{2} + \xi - \sqrt{\left( 1+\xi \right) ^2 + \zeta ^2} \right) , \end{aligned}$$

with $\mathcal {V}_\textrm{G} = \mathcal {Q}_\mathrm {G_B} + \mathcal {R}_\mathrm {G_B}$. As in the ODE case, the residual $\mathcal {R}_{\mathrm {G_B}}$ remains non-negative under the condition $v(x,t)>-1$. This ensures that the string does not fold back on itself, which would be unphysical. As in the ODE case, this constraint cannot be forced to hold a priori. Form (48) is nonetheless included in the analysis since, in contrast to (47), its quadratic and residual parts cleanly separate the linear from the nonlinear dynamics.

Similarly to the ODE case, the following analysis will employ SAV NS with the full geometrically exact potential $\mathcal {V}_\textrm{G}$, and SAV S with residuals $\mathcal {R}_\mathrm {G_A}$ and $\mathcal {R}_\mathrm {G_B}$. These will be referred to as $\textrm{G}$, $\mathrm {G_A}$ and $\mathrm {G_B}$, respectively, henceforth.

### Cubic model (C)

The potential in Eq. (47) is often approximated by a Taylor expansion to obtain simpler forms, such as the form presented by Morse and Ingard [1]. A further simplification consists in neglecting longitudinal motion by imposing $\xi (x,t) = 0\; \forall \; t$ in the approximated potential, resulting in the *cubic* model. For a detailed derivation, see [13, 34]. The resulting potential density is denoted here by $\mathcal {V}_\textrm{C}$, with components:

49a $$\begin{aligned} \mathcal {Q}_{\textrm{C}}&= \frac{T_0}{2} \zeta ^2 + \frac{EI}{2} \chi ^2,\end{aligned}$$

49b $$\begin{aligned} \mathcal {R}_{\textrm{C}}&= \frac{EA - T_0}{8} \zeta ^4 . \end{aligned}$$

The residual component $\mathcal {R}_\textrm{C}$ leads to a cubic force density, hence the name. Again assuming $EA \ge T_0$, the residual $\mathcal {R}_\textrm{C}$ is non-negative.

### Kirchhoff-Carrier model (K)

If, instead of neglecting longitudinal motion in the Taylor-approximated potential, one averages it over the string length, the Kirchhoff–Carrier equation is obtained. The corresponding potential density is denoted $\mathcal {V}_\textrm{K}$, with components:

50a $$\begin{aligned} \mathcal {Q}_\textrm{K}&= \frac{T_0}{2} \zeta ^2 + \frac{EI}{2} \chi ^2,\end{aligned}$$

50b $$\begin{aligned} \mathcal {R}_\textrm{K}&= \frac{EA}{8L} \zeta ^2 \int _0^L\zeta ^2 \textrm{d} x , \end{aligned}$$

which are both semi-positive definite. A full derivation is available in classic papers such as that by Anand [14]. The physical constants are as defined previously.

A summary of the quadratised potentials derived from the potentials described in this section is provided in Appendix C. Models $\mathrm C$ and $\mathrm K$ will be analysed both with SAV NS and S. The split forms will be denoted $\mathrm {C-S}$ and $\mathrm {K-S}$, respectively.

## Spatial discretisation

Spatial discretisation is now performed using the Finite Difference (FD) method. The spatial domain is divided into *N* subintervals of length $h = L/N$, the grid spacing, yielding $N+1$ discretisation points. The continuous functions *u*(*x*, *t*) and *v*(*x*, *t*) are thus approximated at these points by discrete grid functions: $u_l(t) \approx u(lh,t)$ and $v_l(t) \approx v(lh,t)$, where $l = 0, 1, \dots , N$ is an integer index. Backward and forward FD approximations to the first-order spatial derivative are defined by their action on the grid function $u_l$ as:

51 $$\begin{aligned} \delta _{ x+}u_l = \frac{u_{l+1} - u_l}{h}; \quad \delta _{x-}u_l = \frac{u_l - u_{l-1}}{h}, \end{aligned}$$

and apply analogously to $v_l$. Thus, discrete versions of the slope $\zeta$ and strain $\xi$ result: $\zeta _{l-\frac{1}{2}} = \delta _{x-}u_l$; $\xi _{l-\frac{1}{2}} = \delta _{x-}v_l$, defined on an interleaved grid. Approximations to higher-order derivatives are constructed by composition: $\delta _{xx}= \delta _{ x+}\delta _{x-}$ and $\delta _{xxxx}= \delta _{xx}\delta _{xx}$. A discrete version of the approximate curvature $\chi$ is written accordingly as $\chi _l = \delta _{xx}u_l$. A discrete version of the boundary conditions (35) is then:

52 $$\begin{aligned} u_0 = u_N = v_0 = v_N = \chi _0 = \chi _N = 0. \end{aligned}$$

These conditions fix the endpoint values of the grid functions at $l = 0$ and $l = N$, so the spatial index can be restricted to the range $l = 1, \dots , N - 1$. The grid functions can be condensed into the state vectors $\textbf{u}(t), \textbf{v}(t) \in \mathbb {R}^{N-1}$, and the spatial difference operators may be represented as matrices acting on the state vectors. In particular, when incorporating the boundary conditions (52), the backward difference operator becomes a rectangular matrix $\textbf{D}^-\in \mathbb {R}^{N \times (N-1)}$, which acts on the vector $\textbf{u}$ as:

53 $$\begin{aligned} \textbf{D}^- \textbf{u}= \frac{1}{h}([\textbf{u}^\intercal ,0] - [0, \textbf{u}^\intercal ])^\intercal . \end{aligned}$$

An analogous definition applies to the vector $\textbf{v}$, and this yields the slope and strain vectors $\boldsymbol{\zeta }= \textbf{D}^-\textbf{u}$, $\boldsymbol{\xi }= \textbf{D}^-\textbf{v}$. The forward operator is conveniently written: $\mathbb {R}^{(N-1) \times N} \ni \textbf{D}^+= -(\textbf{D}^-)^\intercal$. Note that, due to the boundary conditions (52), one has $\boldsymbol{\zeta }, \boldsymbol{\xi }\in \mathbb {R}^N$, i.e. the slope and strain vectors contain one more element than the state vectors. Second- and fourth-order difference operators are obtained by composition: $\textbf{D}^2 = \textbf{D}^+\textbf{D}^-$; $\textbf{D}^4 = \textbf{D}^2\textbf{D}^2$, in $\mathbb {R}^{(N-1)\times (N-1)}$. The approximate curvature vector is thus written $\boldsymbol{\chi } = \textbf{D}^2\textbf{u}\in \mathbb {R}^{N-1}$. Conveniently, the state vector can be given in discrete form as $\textbf{w}= [\textbf{u}, \textbf{v}]^\intercal \in \mathbb {R}^{2(N-1)\times 1}$.

Spatially discretised versions of the energy functionals defined in Sect. 5 are given here as:

54a $$\begin{aligned} K&\triangleq \sum _{l = 1}^{N-1} h\, \mathcal {K}(\dot{u}_l, \dot{v}_l ) = \frac{\rho A}{2}\sum _{l = 1}^{N-1} h\, ({\dot{u}}_l^2 + {\dot{v}}_l^2) \end{aligned}$$

54b $$\begin{aligned} V&\triangleq \sum _{l = 1}^N h\, \mathcal {V}(\zeta _{l-\frac{1}{2}},\chi _{l}, \xi _{l-\frac{1}{2}}), \end{aligned}$$

and the discrete potential can be further decomposed into a quadratic component and a nonlinear residual:

55 $$\begin{aligned} V = Q + R, \end{aligned}$$

where *Q* and *R* represent the discrete counterparts of $\textrm{Q}$ and $\textrm{R}$, as defined in Eq. (34). Extending variational differentiation to the spatially discrete case, a semi-discrete version of (28) results as:

56 $$\begin{aligned} \nabla _{{\dot{\textbf{w}}}} K = - \nabla _{\textbf{w}} V, \end{aligned}$$

The operator $\nabla _\textbf{w}$ indicates the gradient with respect to the vector $\textbf{w}$, and one has:

57a $$\begin{aligned} \nabla _{{\dot{\textbf{w}}}} K\triangleq &   \rho A \begin{bmatrix} \ddot{\textbf{u}} \\ \ddot{\textbf{v}}\end{bmatrix} = \rho A \ddot{\textbf{w}}; \end{aligned}$$

57b $$\begin{aligned} \nabla _{\textbf{w}} V\triangleq &   \begin{bmatrix}- \textbf{D}^+\nabla _{\boldsymbol{\zeta }} \mathcal {V} + \textbf{D}^2\nabla _{\boldsymbol{\chi }} \mathcal {V}\\ - \textbf{D}^+\nabla _{\boldsymbol{\xi }} \mathcal {V}\end{bmatrix}, \end{aligned}$$

where here $\mathcal {V} = \mathcal {V}(\zeta _{l-\frac{1}{2}},\chi _{l}, \xi _{l-\frac{1}{2}})$ are the potential densities from Sect. 5 evaluated on the grid points, as seen in Eq. (54b). The energy balance is directly encoded as:

58 $$\begin{aligned} \dot{H} = 0, \qquad H = K + V, \end{aligned}$$

and the semi-discrete energy *H* is non-negative provided that the discrete potential is.

### Quadratised equations

A unified quadratised form of (56) is obtained by introducing a switch $\sigma \in \{0,1\}$ and defining the target discrete potential:

59 $$\begin{aligned} \Phi \triangleq \sigma V + (1-\sigma ) R, \end{aligned}$$

where $V=Q+R$. The auxiliary variable is defined with shift $\varepsilon >0$ as:

60 $$\begin{aligned} \psi \triangleq \sqrt{2\Phi +\varepsilon }, \qquad \textbf{g}\triangleq \nabla _\textbf{w}\psi = \frac{\nabla _\textbf{w}\Phi }{\sqrt{2\Phi +\varepsilon }}. \end{aligned}$$

The discrete chain rule, serving as the update equation for $\psi$, reads:

61 $$\begin{aligned} {{\dot{\psi }}} = h\,\textbf{g}^\intercal {{\dot{\textbf{w}}}}, \end{aligned}$$

so that (56) becomes:

62a $$\begin{aligned} \rho A \ddot{\textbf{w}}&= -(1-\sigma )\,\nabla _\textbf{w}Q - \psi \textbf{g}, \end{aligned}$$

62b $$\begin{aligned} {{\dot{\psi }}}&= h\,\textbf{g}^\intercal {{\dot{\textbf{w}}}} . \end{aligned}$$

The quadratic gradient is obtained by applying (57b) to *Q* alone, yielding:

63 $$\begin{aligned} \nabla _\textbf{w}Q = {\textbf{K}} \textbf{w}, \end{aligned}$$

where ${\textbf{K}}$ is the positive semi-definite matrix arising from spatial discretisation of $\mathcal {L}$. For $\sigma =1$, the quadratic term vanishes and the full potential is incorporated into $\psi$. For $\sigma =0$, only the residual is quadratised and ${\textbf{K}} \textbf{w}$ appears explicitly.

The discrete energy balance remains that of (58), and the quadratised energy can be written in unified form as:

64 $$\begin{aligned} H = K + (1-\sigma )\,Q + \frac{\psi ^2}{2}, \end{aligned}$$

which is non-negative.

A summary of the spatially discrete expressions for *Q* and *R* is provided in Appendix C.

## Time discretisation

In discrete time, $\textbf{w}(t)$ is approximated by the sequence $\textbf{w}^n \approx \textbf{w}(nk)$. The difference operators are defined as in Sect. 3.

### SAV solver

A unified distributed SAV scheme is obtained directly from (62) by retaining the switch $\sigma \in \{0,1\}$:

65a $$\begin{aligned} \delta _{tt}\textbf{w}^n&= -(1-\sigma )\,\textbf{K}\textbf{w}^n - \mu _{ t+}\psi ^{n-\frac{1}{2}} \textbf{g}^n, \end{aligned}$$

65b $$\begin{aligned} \delta _{ t+}\psi ^{n-\frac{1}{2}}&= h (\textbf{g}^n)^\intercal \delta _{t\cdot }\textbf{w}^n, \end{aligned}$$

with $\textbf{g}^n = \textbf{g}(\textbf{w}^n)$ and where $\textbf{K}$ is the matrix representation of the quadratic operator. Expanding (65a) yields the update:

66 $$\begin{aligned} \textbf{A}^n \textbf{w}^{n+1} = \textbf{B}\textbf{w}^n + \textbf{C}^n \textbf{w}^{n-1} - \frac{k^2 h}{\rho A} \textbf{g}^n \psi ^{n-\frac{1}{2}}, \end{aligned}$$

where the matrices read:

67a $$\begin{aligned} \textbf{A}^n&\triangleq \textbf{I} + \frac{k^2 h}{4\rho A} \textbf{g}^n (\textbf{g}^n)^\intercal , \end{aligned}$$

67b $$\begin{aligned} \textbf{B}&\triangleq 2\textbf{I} + \frac{k^2}{\rho A}(1-\sigma )\textbf{K} \end{aligned}$$

67c $$\begin{aligned} \textbf{C}^n&\triangleq -\textbf{I} + \frac{k^2 h}{4\rho A} \textbf{g}^n (\textbf{g}^n)^\intercal . \end{aligned}$$

Here $\textbf{I}\in \mathbb {R}^{2(N-1)\times 2(N-1)}$ is the identity matrix. The matrix $\textbf{A}^n$ has the form of the identity plus a rank-1 perturbation and can therefore be inverted efficiently using the Sherman–Morrison formula [32].

For $\sigma =1$ the quadratic term vanishes, recovering the non-split formulation. For $\sigma =0$ the quadratic contribution is retained explicitly.

The discrete energy balance follows as in the ODE case, yielding:

$$ \delta _{ t+}{\mathfrak {h}}^{n-\frac{1}{2}} = 0, $$

with energy:

$$ {\mathfrak {h}}^{n-\frac{1}{2}} = {\mathfrak {K}}^{n-\frac{1}{2}} + (1-\sigma )\,{\mathfrak {Q}}^{n-\frac{1}{2}} + \frac{\bigl (\psi ^{n-\frac{1}{2}}\bigr )^2}{2}. $$

Non-negativity of the quadratic contribution requires the same condition seen in (26):

68 $$\begin{aligned} 4 - k^2 (1-\sigma )\lambda _{\max }({\textbf{K}}) > 0, \end{aligned}$$

which reduces to the standard CFL-type condition in the split case and is void for $\sigma =1$. Under this condition, the scheme is energy-conservative and stable.

**Table 1:** Sampling rate $f_s$ (MHz) and corresponding time step $k = 1/f_s$ ($\mu$s) as a function of the oversampling exponent *a*, with $f_s = 44100 \cdot 2^{a}$ Hz.

*a*	0	1	2	3	4	5	6	7	8	9	10
$f_s$ [MHz]	$4.41\!\cdot \!10^{-2}$	$8.82\!\cdot \!10^{-2}$	0.18	0.35	0.71	1.41	2.82	5.64	11.3	22.6	45.2
*k* [$\mu$s]	22.7	11.3	5.67	2.83	1.42	0.71	0.35	0.18	0.088	0.044	0.022

## Numerical results

A numerical test similar to that seen for the ODE is performed here. The string models introduced in Sects. 5.2, 5.3, and 5.4 are simulated with SAV, at decreasing time-steps, using the potential gradients provided in Table 7. For each string model, a reference solution is first generated using a benchmark integrator from the literature, run with a very small time step. In the case of model G, the Störmer–Verlet (SV) integrator is adopted. Although an energy-preserving scheme exists for this model [19], its computational cost makes it impractical for the sample rates considered here. For models C and K, two energy-preserving and convergent schemes available in the literature are instead employed. The errors between the benchmark and SAV solutions are then evaluated as:

69 $$\begin{aligned} E(t_E,x_E) = {\hat{u}}^{{\hat{n}}_E}_{{\hat{l}}_E} - u^{n_E}_{l_E}, \end{aligned}$$

where $$x_E$$ is a pre-defined output location, and $${\hat{u}}^{{\hat{n}}_E}_{{\hat{l}}_E}$$ denotes the reference solution computed at $${\hat{n}}_E \triangleq t_E/{\hat{k}}$$, $${\hat{l}}_E \triangleq x_E/{\hat{h}}$$, with $${\hat{h}}$$ representing the spatial step of the reference solution. Similarly, $$ u^n_l$$ is computed at $$n_E \triangleq t_E/ k$$, $$l_E \triangleq x_E/ h$$. All string simulations use a fixed evaluation time of $$t_E = 0.06$$: at the highest sample rates, computational cost precludes the use of longer simulation windows. For the transverse displacement, it is set: $$x_E = L/2$$, while for the longitudinal displacement $$x_E = L/4$$. The number of spatial points is always adjusted to ensure that these locations coincide exactly with grid nodes. Consequently, the space–time discretisation steps are recalculated according to the stability condition in use. Tests are repeated using the potential shifts $$\varepsilon _a$$ and $$\varepsilon _b$$ defined in Sect. 4. To assess convergence of the auxiliary variable, reference values are obtained from the benchmark solutions using the definition in Eq. (60), with both values of $$\varepsilon $$. The error is then computed at the staggered time instant $$t_E -\tfrac{k}{2}$$.

All simulations employ physical parameters representative of a steel string: $\rho = 8050$ kg/$\hbox {m}^3$, $A = 3.97 \times 10^{-7}$ $\hbox {m}^2$, $T_0 = 75$ N, and $E = 174$ GPa, with a string length $L=1$ m. The strings are initialised with a maximum amplitude of 2 mm, both in the first vibration mode (henceforth “MOD”), as well as with a raised cosine centred at the string midpoint with a width equal to $25\%$ of the string length (henceforth “RC”). A base audio sampling rate of 44100 Hz is used, which is then increased with an integer OF expressed as a power of 2, as seen for the ODE. The SAV schemes are run with $a \in [1,9]$, while the benchmark schemes use $a=10$. Table 1 presents the resulting sample rates, note that these are above the MHz range for a ≥ 5.

### Model G

For the geometrically exact model, two stability conditions arise, one related to the longitudinal wave speed and the other to the transverse wave speed. The first is a pure CFL condition on the longitudinal wave speed:

70 $$\begin{aligned} h \ge \sqrt{\frac{E}{\rho }}k \triangleq h_\textrm{L} . \end{aligned}$$

The transverse condition, on the other hand, is not a pure CFL condition, as a consequence of the direct discretisation of the fourth spatial derivative:

71 $$\begin{aligned} h \ge \sqrt{\frac{T_0 k^2 + \sqrt{\left( T_0k^2 \right) ^2 + 16\rho A EI k^2}}{2\rho A}} \triangleq h_\textrm{T} ; \end{aligned}$$

**Fig. 3:**
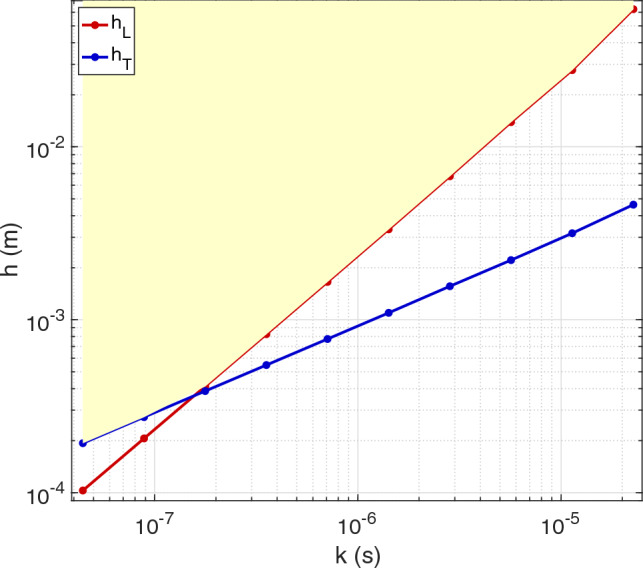
Values of $h_\textrm{L}$ and $h_\textrm{T}$ computed from Eqs. (70) and (71), as functions of *k* corresponding to $a \in [2,10]$. The yellow regions indicate the stability region of the quadratic part for *h*

For the linear part of the split integrator to remain stable and avoid instability, the spatial step size must be at least as large as the greater of $$h_\textrm{L}$$ and $$h_\textrm{T}$$. Figure 3 displays $$h_\textrm{L}$$ and $$h_\textrm{T}$$, computed using the physical parameters listed above and plotted against *k*. A logarithmic scale is used for clarity. The yellow region indicates the quadratic stability region where *k*-*h* pairs can be selected. The minimum admissible value of *h* corresponds to the pointwise maximum between the two curves, denoted $$h_\textrm{MAX}$$. For the string parameters used here, the two curves intersect, and one has $$h_{\textrm{MAX}} = h_\textrm{L}$$ for $$a \le 8$$, and $$h_{\textrm{MAX}} = h_\textrm{T}$$ for $$ a> 8$$. The spatial step is always set 1% above the stability limit to avoid further spurious numerical artefacts, as seen in e.g. [7]: $$h = 1.01h_{\textrm{MAX}} \triangleq {\bar{h}}$$.

Since the stability properties of the SV method applied to the geometrically exact nonlinear residual are not established, the space and time discretisation parameters are selected with respect to the quadratic component only. Nevertheless, the integrator remained stable under the chosen initial conditions, provided that $h \ge h_{\textrm{MAX}}$. The validity of the benchmark solutions is assessed via Table 2, which reports the solution values at $t_E = 0.06$ s for increasing oversampling. Forms $\textrm{G}/\mathrm {G_A}$ (these are identical for the SV solver) and $\hbox {G}_\textrm{B}$ yield identical solutions up to 12 decimal places, confirming their equivalence. Furthermore, the SV solutions converge up to the 7th decimal place for the transverse motion and the 8th for the longitudinal motion. The benchmark solutions used to validate SAV correspond to $a=10$.

#### SAV convergence test

**Fig. 4:**
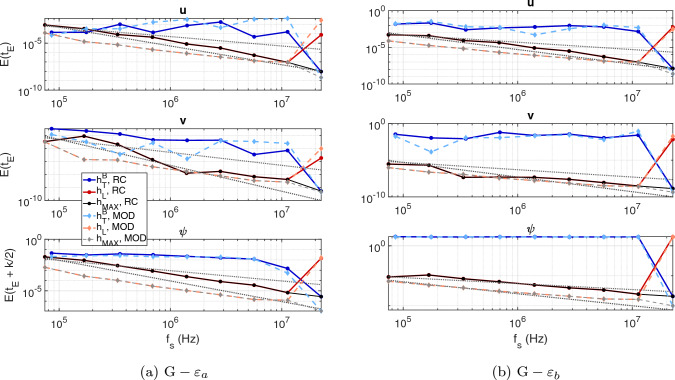
Convergence results for the geometrically exact string, Form $\textrm{G}$

**Fig. 5:**
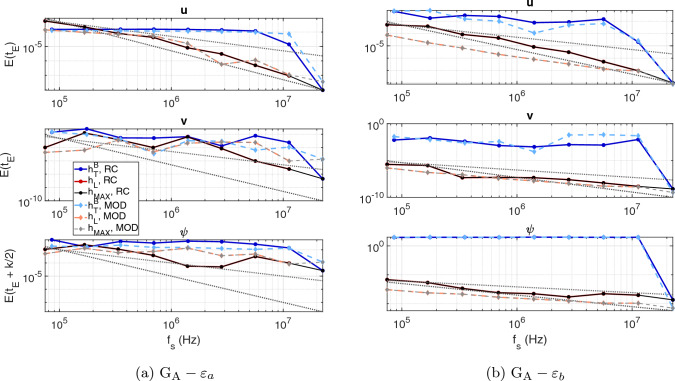
Convergence results for the geometrically exact string, Form $\mathrm {G_A}$

**Fig. 6:**
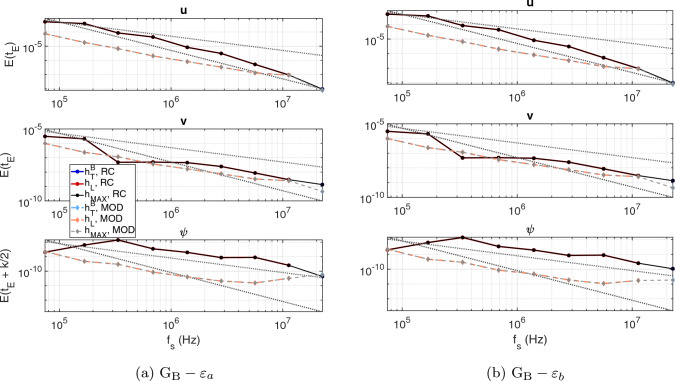
Convergence results for the geometrically exact string, Form $\mathrm {G_B}$

All tests are run using $h = {\bar{h}}$. Results are shown in Figs. 4, 5, and 6. For each potential type, the two panels display results for two different shift constants. Each panel contains results for both initialisation types, along with two reference dotted lines of slope $-1$ and $-2$. Errors are plotted against the sample rate rather than the spatial step size, the most common choice. As a consequence, the rate of convergence is expected to be lower than two, due to the direct discretisation of the stiffness term. This representation was chosen for visual clarity.

**Table 2:** Solution values at $t_E=0.06$ s, computed with SV for Forms $\textrm{G}$/$\mathrm {G_A}$ and Form $\mathrm {G_B}$, initialised with MOD and RC, across 2 increasing OF values expressed via the exponent *a*. “Transv.” and “Long.” denote the transverse and longitudinal solutions, respectively. The bold entries indicate the benchmark values used for SAV testing

Form $\textrm{G}$/$\mathrm {G_A}$	MOD		RC	
a	Transv	Long	Transv	Long
3	−0.001542536764	0.000000455045	−0.000067010490	−0.000004989832
4	−0.001537884027	0.000000534644	−0.000108258834	−0.000005085885
5	−0.001536638849	0.000000554477	−0.000143945383	−0.000005081376
6	−0.001536158952	0.000000563468	−0.000149147281	−0.000005060437
7	−0.001535953014	0.000000567741	−0.000151706166	−0.000005044582
8	−0.001535913124	0.000000568515	−0.000152133566	−0.000005032951
9	−0.001535830208	0.000000570587	−0.000152218477	−0.000005037279
**10**	**−0.001535822314**	**0**.**000000571029**	**−0.000152228140**	**−0.000005035934**

Form $G_{B}$	MOD		RC	
a	Transv	Long	Transv	Long
3	−0.001542536880	0.000000454636	−0.000067010580	−0.000004987832
4	−0.001537884054	0.000000534567	−0.000108258848	−0.000005085587
5	−0.001536638855	0.000000554461	−0.000143945391	−0.000005081310
6	−0.001536158954	0.000000563464	−0.000149147283	−0.000005060421
7	−0.001535953015	0.000000567740	−0.000151706168	−0.000005044578
8	−0.001535913125	0.000000568515	−0.000152133566	−0.000005032950
9	−0.001535830208	0.000000570587	−0.000152218477	−0.000005037279
**10**	**−0.001535822314**	**0**.**000000571029**	**−0.000152228140**	**−0.000005035934**

Given the unconditional stability of scheme $\textrm{G}$, the choice of spatial step size is irrelevant to stability. However, for this scheme (Fig. 4), convergence is observed only when $h_\textrm{MAX}$ is used, even though the scheme remains stable below this threshold. Specifically, for $a \le 8$ the scheme with $h_\textrm{L}$ converges while that with $h_\textrm{T}$ does not; at $a = 9$ the behaviour reverses. The only consistent path to convergence is therefore to adopt $h_\textrm{MAX}$, switching from $h_\textrm{L}$ to $h_\textrm{T}$ once *a* exceeds 8 (as shown in Fig. 3), in which case all three monitored quantities converge. This holds for both values of $\varepsilon$; however, as shown in the first row of Tab. 3, a larger shift constant yields a substantial improvement in the error of $\psi$.

**Table 3:** Convergence errors *E* for SAV schemes $\textrm{G}$, $\mathrm {G_A}$ and $\mathrm {G_B}$ when $a=9$

Scheme	$\varepsilon$	MOD			RC		
		*u*	*v*	$\psi$	*u*	*v*	$\psi$
$\textrm{G}$	$\varepsilon _a$	$2.18\times 10^{-9}$	$4.37\times 10^{-10}$	$1.11\times 10^{-7}$	$9.39\times 10^{-9}$	$5.17\times 10^{-10}$	$2.74\times 10^{-6}$
	$\varepsilon _b$	$2.42\times 10^{-9}$	$4.35\times 10^{-10}$	$9.84\times 10^{-11}$	$1.30\times 10^{-8}$	$1.34\times 10^{-9}$	$3.76\times 10^{-9}$
$\mathrm {G_A}$	$\varepsilon _a$	$3.63\times 10^{-8}$	$1.35\times 10^{-7}$	$1.17\times 10^{-4}$	$9.49\times 10^{-9}$	$4.46\times 10^{-9}$	$2.62\times 10^{-5}$
	$\varepsilon _b$	$7.89\times 10^{-9}$	$4.42\times 10^{-10}$	$1.65\times 10^{-11}$	$9.66\times 10^{-9}$	$1.35\times 10^{-9}$	$4.50\times 10^{-10}$
$\mathrm {G_B}$	$\varepsilon _a$	$7.89\times 10^{-9}$	$4.42\times 10^{-10}$	$5.81\times 10^{-11}$	$9.66\times 10^{-9}$	$1.35\times 10^{-9}$	$4.30\times 10^{-11}$
	$\varepsilon _b$	$7.89\times 10^{-9}$	$4.42\times 10^{-10}$	$1.85\times 10^{-11}$	$9.66\times 10^{-9}$	$1.35\times 10^{-9}$	$1.08\times 10^{-10}$

For scheme $\mathrm {G_A}$ (Fig. 5), the separation between linear and nonlinear dynamics is incomplete. When computed with $h_\textrm{L}$, the scheme becomes unstable for $a > 8$, yielding no solution, whereas with $h_\textrm{T}$ it remains stable throughout. With $h_\textrm{MAX}$ and $\varepsilon _a$, convergence is observed for *u* under MOD initialisation, but not for *v* and $\psi$; under RC initialisation, *v* also exhibits a clearer convergence trend. Using the larger shift constant $\varepsilon _b$ resolves this: as shown in the second row of Table 3, clear convergence is recovered for all three quantities under MOD initialisation. Under RC initialisation, *u* and *v* were already converging, but a substantial improvement in $\psi$ is still observed.

Finally, in scheme $\mathrm {G_B}$ (6), where there is a clear separation between linear and nonlinear energy, using *h* below the stability limit causes the scheme to explode, as expected. When $h_\textrm{MAX}$ is used, convergence is observed for all quantities and both initialisations, and the results are insensitive to the choice of $\varepsilon$. This is confirmed by the error values in the third row of Table 3.

Note that plotting the errors against the sample rate rather than the spatial step allows for a direct comparison between cases governed by different stability conditions.

**Fig. 7:**
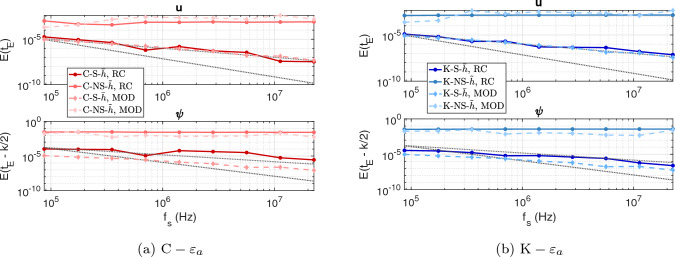
Convergence results for strings C and K with potential shift $\varepsilon _a$

### Model C

For the cubic model, a reference integrator proposed by Bilbao [13] is used, given by:

72 $$\begin{aligned} \begin{aligned} \rho A\delta _{tt}\textbf{u}^n&= T_0\textbf{D}^2\textbf{u}^n - EI \textbf{D}^4\textbf{u}^n +\\&\frac{EA - T_0}{2} \textbf{D}^+\boldsymbol{\zeta }^2 \mu _{t\cdot }\boldsymbol{\zeta }. \end{aligned} \end{aligned}$$

Since the benchmark scheme is convergent, no additional validation test is required for the reference solution, which is obtained with $a=10$. Here, $h_\textrm{MAX} = h_\textrm{T}$ and it is employed $h = {\bar{h}}$ throughout.

#### SAV convergence test

A first test is performed with $h={\bar{h}}$ and $\varepsilon _a$. For brevity, only the split-SAV results are reported in Fig. 7, panel 7a, as the NS and S algorithms produce identical convergence rates, and similar error values. Results using $\varepsilon _b$, not shown here, yield identical convergence rates but improve the accuracy of the auxiliary variable. An analysis of the Jacobian $\textrm{J}{{\textbf{g}}}$ (not shown here) shows a qualitative difference between the two formulations: in the NS case, as in the geometrically exact string, $\textrm{J}{{\textbf{g}}}$ becomes unbounded as $u \rightarrow 0$. In the split case, instead, it may be proved that $\textrm{J}{{\textbf{g}}}=0$ at $u=0$ and, although it can grow as $\epsilon \rightarrow 0^+$ for *u* in a neighbourhood of 0, it remains bounded.

A second test is then run with SAV NS, using $h = 0.99\,h_\textrm{T} \triangleq {\tilde{h}}$ (i.e., slightly below the stability condition of the split version). From Fig. 7a, it may be observed that, although the scheme is unconditionally stable, convergence is not observed in this case.

### Model K

As a reference, another convergent, unconditionally stable integrator from Bilbao [7] is here used:

73 $$\begin{aligned} \begin{aligned} \rho A\delta _{tt}\textbf{u}^n&= T_0\textbf{D}^2\textbf{u}^n - EI \textbf{D}^4\textbf{u}^n+ \\&\frac{EAh}{2L} \textbf{D}^+\boldsymbol{\zeta }\mu _{ t+}\left[ (\boldsymbol{\zeta }^n)^\intercal \boldsymbol{\zeta }^{n-1}\right] . \end{aligned} \end{aligned}$$

The benchmark solution was computed with $a=10$, with $h = {\bar{h}}$.

#### SAV convergence test

A first test with $h = {\bar{h}}$ is performed using both NS and S SAV. Similarly to the cubic case, convergence is observed for both algorithms, and only the results for the S form are shown, in Fig. 7b

As for string C, a second test carried out with SAV NS using $h = {\tilde{h}}$ shows that, even though the integrator remains stable, it does not converge when the time step lies below the stability condition of the split version, matching the results obtained with the other models.

## Discussion

Although the SAV framework guarantees energy conservation and unconditional stability, the present results show that these properties do not automatically ensure numerical convergence in wave-type problems. Instead, convergence is strongly influenced by three factors: the spatial sampling step, the partitioning of the quadratic and residual components of the potential energy, and the potential shift constant.

NS schemes achieve convergence only when the spatial step satisfies $h \ge h_{\textrm{MAX}}$, i.e., when *h* lies at or above the stability threshold imposed by the quadratic component of the corresponding split formulation. Hence, despite formal unconditional stability, reducing *h* below this threshold produces unreliable solutions that fail to converge, regardless of how finely the time step is refined; this behaviour is observed consistently across all string types considered.

When remaining within stability limits, convergence is influenced by the form of the potential treated with SAV. For strings C and K, no meaningful difference in convergence rate or accuracy is observed between the NS and S formulations. For the geometrically exact string, the numerical results are consistent with the mathematical analysis carried out for the ODE in Sect. 4, in particular concerning the convergence of the auxiliary variable $\psi$. It should be noted, however, that the simulation windows accessible at the sample rates considered here are relatively short. As suggested by the ODE tests, the accumulated error over longer simulation windows would most likely degrade convergence further in models G, $\mathrm {G_A}$ and the NS formulations of strings C and K, where the Jacobian of $\textbf{g}$ grows near the equilibrium.

The influence of the shift constant $\varepsilon$ on convergence is confirmed by the results, corroborating initial observations by Zama et al. [43]. The present study makes it possible to relate this influence directly to the behaviour of the Jacobian of $\textbf{g}$. In schemes where the SAV potential vanishes at equilibrium, yet its gradient or Hessian does not (C-NS, K-NS, G, $\hbox {G}_\textrm{A}$), increasing $\varepsilon$ substantially improves the accuracy of the auxiliary variable $\psi$ and, in borderline cases, can restore convergence of the displacement components. Scheme $\hbox {G}_\textrm{B}$, by contrast, yields results that are essentially insensitive to $\varepsilon$, consistent with the theoretical prediction that the Lipschitz constant of $\textbf{g}$ is insensitive to $\varepsilon$ in this case. Scheme $\hbox {G}_\textrm{B}$ remained stable throughout all simulations, despite the requirement $v > -1$ for the non-negativity of the residual $\mathcal {R}_{\mathrm {G_B}}$. This constraint cannot be guaranteed a priori from energy arguments alone, and longer simulations or larger initial amplitudes may cause it to be violated, potentially compromising the scheme. Such regimes, however, are unphysical: $v \le -1$ corresponds to a negative local stretch, i.e. a loss of orientation-preservation in which material points fold back on themselves.

For scheme C-S, increasing $\varepsilon$ improves the accuracy of the auxiliary variable, yet clear convergence rates are obtained even for small values of the shift: although the Jacobian of $\textbf{g}$ grows as $\varepsilon \rightarrow 0$, it remains bounded, so that the error constants in the convergence estimate are merely increased rather than rendered unbounded. In scheme K-S, on the other hand, no shift constant is required, and the Jacobian is bounded independently. Table 4 provides a summary of the main observed results.

As noted in Sect. 1, several techniques have indeed been proposed to regularise the behaviour of the SAV auxiliary variable, including drift correction [42] and constraint enforcement [35, 41]. However, the purpose of the present work is to investigate the convergence properties of SAV in its original, unmodified form. If SAV converges, the auxiliary variable naturally recovers its correct behaviour as the sample rate is increased, without the need for additional correction. A convergence analysis of SAV augmented with regularisation techniques is left for future work.

**Table 4:** Summary of convergence results for each string model and SAV formulation

Model	SAV NS	SAV S
Geometrically exact	Converges only for $h \ge h_{\textrm{MAX}}$. Jacobian unbounded as $\varepsilon \rightarrow 0^+$; larger error for small $\varepsilon$	*Form G*$_\textrm{A}$: Incomplete linear-nonlinear split. Jacobian unbounded as $\varepsilon \rightarrow 0^+$; larger error for small $\varepsilon$
		*Form G*$_\textrm{B}$: Complete linear-nonlinear split. Jacobian bounded; insensitive to $\varepsilon$
Cubic	Converges only for $h \ge h_{\textrm{MAX}}$. Complete linear-nonlinear split. Jacobian unbounded as $\varepsilon \rightarrow 0^+$; larger error for small $\varepsilon$	Complete linear-nonlinear split. Jacobian bounded at equilibrium but grows as $\varepsilon \rightarrow 0^+$ in a neighbourhood of 0; larger error for small $\varepsilon$
Kirchhoff–Carrier	Converges only for $h \ge h_{\textrm{MAX}}$. Jacobian unbounded as $\varepsilon \rightarrow 0^+$; larger error for small $\varepsilon$	Jacobian bounded independently of $\varepsilon$; no shift required

## Conclusions

This work presented a systematic numerical convergence study of the Scalar Auxiliary Variable (SAV) method applied to three stiff string models with geometric nonlinearities: the geometrically exact model, the cubic, and the Kirchhoff–Carrier models. A unified continuous formulation was developed, together with the quadratisation approach in both its non-split and split forms. Corresponding finite-difference discretisations were introduced, and benchmark integrators were defined for each model: the Störmer–Verlet (SV) scheme for the geometrically exact string, and unconditionally stable reference schemes for the cubic and Kirchhoff–Carrier cases. Convergence tests compared these benchmark solutions with SAV-based results at progressively increasing sample rates, assessing both displacement components and the auxiliary variable.

For all models, the non-split formulation achieves convergence only when the spatial step respects the stability bound associated with the quadratic component of the corresponding split formulation, despite the unconditional stability of the scheme. Furthermore, NS schemes are characterised by an unbounded Jacobian of the SAV nonlinear gradient near equilibrium, which, following the analysis of the ODE problem, implies that the global error constant grows without bound as $\varepsilon \rightarrow 0^+$, making accuracy strongly dependent on the choice of the potential shift $\varepsilon$.

The split formulation performs reliably only when the quadratic–residual energy decomposition aligns naturally with the system’s physical linear–nonlinear separation, as is the case for the cubic and Kirchhoff-Carrier strings, and for Form B of the geometrically exact string. In these cases, the Jacobian of the SAV nonlinear gradient is bounded, and convergence is achieved with well-defined rates. Among these, Form B of the geometrically exact string and the Kirchhoff–Carrier model are insensitive to the shift constant $\varepsilon$, while the cubic model exhibits a Jacobian norm that grows as $\varepsilon \rightarrow 0^+$ in a neighbourhood of the equilibrium (and vanishes on the equilibrium) yet remains bounded throughout, resulting in a larger but finite global error.

These findings suggest that the SAV approach offers limited practical benefit for the geometrically exact string, particularly in the context of real-time simulation for musical acoustics. Reliable results require extremely fine temporal resolution, since the mesh size required for convergence is much larger than the mesh size associated with the transverse wave speed, negating the potential advantage of an explicit integrator. Indeed, one motivation for considering the non-split formulation was precisely to circumvent the stability constraint on the spatial step imposed by the quadratic component of the split potential, allowing finer mesh sizes and improving accuracy; the present results demonstrate that this is not achievable without sacrificing convergence. Furthermore, the split formulation offering the best convergence behaviour, Form B, relies on the assumption $v > -1$, which, despite encoding a natural physical constraint of the geometrically exact model, cannot be guaranteed a priori via energy arguments. The Kirchhoff-Carrier model, which already admits an unconditionally stable, convergent, and explicit integrator without recourse to SAV, represents a case where the method offers no additional benefit. The cubic model remains the most suitable context for the SAV formulation, consistent with prior work demonstrating improved performance through regularisation and modal variants that reduce numerical dispersion. Future work will focus on quantifying the discrepancy between reference solutions obtained from the geometrically exact model and those produced by its common approximations in the context of musical string synthesis. The aim is to determine whether the additional complexity of the geometrically exact formulation is justified by perceptible or practically relevant improvements in accuracy.

## Data Availability

No datasets were generated or analysed during the current study.

## Appendix A Well-posedness of the Cauchy problem

Define $\textbf{p} \triangleq {{\dot{\textbf{w}}}}$ and write the unified system (11) in first-order form:

A1a $$\begin{aligned} {{\dot{\textbf{w}}}}&= \textbf{p},\end{aligned}$$

A1b $$\begin{aligned} \dot{\textbf{p}}&= -(1\!-\!\sigma )\,\textbf{K}\textbf{w}- \psi \,\textbf{g},\end{aligned}$$

A1c $$\begin{aligned} {{\dot{\psi }}}&= \textbf{g}^\intercal \textbf{p}. \end{aligned}$$

Non-negativity of (13) gives $\Vert \textbf{p}\Vert _2 \le \sqrt{2H_0}$ and $|\psi | \le \sqrt{2H_0}$, with $H_0 = H|_{t=0}$. In continuous time, the norm of $\textbf{w}$ is bounded via the quadratic part of the potential energy, regardless of whether this is absorbed in the definition of $\psi$. From (2) and (4), and using $R \ge 0$ (which holds for the non-split system, split form A and split form B provided $v>-1$), one has

A2 $$\begin{aligned} Q \le V \le H_0 \;\Longrightarrow \; \textbf{w}^\intercal \textbf{K} \textbf{w}\le 2 H_0, \end{aligned}$$

and since $\textbf{K}$ is positive definite, this yields

A3 $$\begin{aligned} \Vert \textbf{w}\Vert _2 \le \sqrt{2 H_0 / \lambda _{\min }(\textbf{K})} = \sqrt{2H_0}, \end{aligned}$$

where the last equality uses $\lambda _{\min }(\textbf{K}) = \min (1,\alpha ) = 1$, since $\alpha \ge 1$. Define the admissible domain

$$ \mathcal {D} = \bigl \{ (\textbf{w}, \textbf{p}, \psi ) \;:\; v > -1,\; \Vert \textbf{w}\Vert \le \sqrt{2H_0}, \bigr . $$

$$ \bigl . \Vert \textbf{p}\Vert \le \sqrt{2H_0},\; |\psi | \le \sqrt{2H_0} \bigr \}. $$

The right-hand side of (A1) defines a vector field $\textbf{f}(\textbf{x})$ over $\textbf{x} \triangleq (\textbf{w}, \textbf{p}, \psi )$. Using the norm $\Vert \textbf{x}\Vert = \Vert \textbf{w}\Vert _2 + \Vert \textbf{p}\Vert _2 + |\psi |$, standard manipulations (triangle inequality; adding and subtracting $\textbf{g}_2\psi _1$) yield, with $\textbf{g}_i \triangleq \textbf{g}(\textbf{w}_i)$,

$$ \begin{aligned}&\Vert \textbf{f}(\textbf{x}_1) - \textbf{f}(\textbf{x}_2)\Vert \\&\quad \le (1\!-\!\sigma )\,\lambda _{\max }(\textbf{K})\, \Vert \textbf{w}_1 - \textbf{w}_2\Vert _2 \\&\qquad + |\psi _1|\,\Vert \textbf{g}_1 - \textbf{g}_2\Vert _2 \\&\qquad + \Vert \textbf{g}_2\Vert _2\,|\psi _1 - \psi _2| \\&\qquad + \Vert \textbf{g}_1 - \textbf{g}_2\Vert _2\,\Vert \textbf{p}_1\Vert _2 \\&\qquad + (1 + \Vert \textbf{g}_2\Vert _2)\, \Vert \textbf{p}_1 - \textbf{p}_2\Vert _2. \end{aligned} $$

If $\textbf{g}$ is Lipschitz on $\mathcal {D}$ with constant $\mathcal {C}_g$, one obtains $\Vert \textbf{f}(\textbf{x}_1) - \textbf{f}(\textbf{x}_2)\Vert \le \mathcal {C}\, \Vert \textbf{x}_1 - \textbf{x}_2\Vert$, with

$$ \begin{aligned} \mathcal {C} = \max \bigl (&(1\!-\!\sigma )\,\lambda _{\max }(\textbf{K}) \\&+ 2\sqrt{2 H_0}\,\mathcal {C}_g, \;\;1 + G_0 \bigr ), \end{aligned} $$

where $G_0 \triangleq \sup _{\textbf{w}\in \mathcal {D}} \Vert \textbf{g}\Vert _2$. The Picard–Lindelöf theorem then guarantees local existence and uniqueness. For $\sigma = 1$ (NS) the contribution of $\textbf{K}$ vanishes; for $\sigma = 0$ (S) it is fully retained.

## Appendix B Convergence analysis of the discrete SAV

### First-order form and initialisation

A first-order form of the discrete unified scheme (21) reads

B4a $$\begin{aligned} \delta _{ t+}\textbf{w}^n&= \textbf{p}^{n+\frac{1}{2}}, \end{aligned}$$

B4b $$\begin{aligned} \delta _{ t+}\textbf{p}^{n-\frac{1}{2}}&= -(1\!-\!\sigma )\,\textbf{K}\textbf{w}^n \nonumber \\&\quad - \textbf{g}^n\,\mu _{ t+}\psi ^{n-\frac{1}{2}},\end{aligned}$$

B4c $$\begin{aligned} \delta _{ t+}\psi ^{n-\frac{1}{2}}&= (\textbf{g}^n)^\intercal \, \mu _{ t+}\textbf{p}^{n-\frac{1}{2}}. \end{aligned}$$

Second-order initialisation from $\textbf{w}_0$, $\textbf{p}_0$ proceeds by setting $\textbf{w}^0 = \textbf{w}_0$, $\textbf{p}^0 = \textbf{p}_0$, $\psi _0 = \sqrt{2\Phi (\textbf{w}_0)+\varepsilon }$, $\textbf{g}_0 = \nabla \Phi (\textbf{w}_0)/\psi _0$, together with

$$ \begin{aligned} \dot{\textbf{p}}_0&= -(1\!-\!\sigma )\,\textbf{K}\textbf{w}_0 - \textbf{g}_0\psi _0,\\ {{\dot{\psi }}}_0&= \textbf{g}_0^\intercal \textbf{p}_0, \end{aligned} $$

and the half-step values $\textbf{p}^{\frac{1}{2}} = \textbf{p}_0 + \tfrac{k}{2}\dot{\textbf{p}}_0$ and $\psi ^{\frac{1}{2}} = \psi _0 + \tfrac{k}{2}{{\dot{\psi }}}_0$.

Since $\delta _{t+}\mathfrak {h}^{n-\frac{1}{2}} = 0$, the discrete energy is exactly conserved. Denote its constant value $\mathfrak {H}^{\frac{1}{2}} \triangleq \mathfrak {h}^{\frac{1}{2}}$. Under the stability condition (26), this yields the uniform bounds

B5 $$\begin{aligned} \begin{aligned} \Vert \textbf{p}^{n+\frac{1}{2}}\Vert _2&\le C_p \triangleq \sqrt{\frac{8\,\mathfrak {H}^{\frac{1}{2}}}{4 - (1\!-\!\sigma )\,k^2\, \lambda _{\max }(\textbf{K})}}, \\ |\psi ^{n+\frac{1}{2}}|&\le C_\psi \triangleq \sqrt{2\,\mathfrak {H}^{\frac{1}{2}}}, \end{aligned} \end{aligned}$$

which hold for all *n*. Following (B4a), one obtains $\Vert \textbf{w}^n\Vert \le \Vert \textbf{w}_0\Vert + nk\,C_p$, so that $\textbf{w}^n$ grows at most linearly. This bound holds unconditionally for both $\sigma = 0$ and $\sigma = 1$, and is sufficient for the convergence argument that follows. Note that $C_p$ is always a positive, real constant when the stability condition (26) is respected.

### Truncation error

Let $t_n = nk$ and $t_{n+\frac{1}{2}} = t_n + k/2$. Substituting the exact solution into (B4) and expanding via the standard identities

$$ \begin{aligned} \delta _{ t+}a(t_n)&= {\dot{a}}(t_{n+\frac{1}{2}}) + \tfrac{k^2}{24}\,a^{(3)}(\xi _n), \\ \mu _{ t+}a(t_{n-\frac{1}{2}})&= a(t_n) + \tfrac{k^2}{8}\,a''(\eta _n), \end{aligned} $$

with $\xi _n \in (t_n, t_{n+1})$, $\eta _n \in (t_{n-\frac{1}{2}}, t_{n+\frac{1}{2}})$, and using (A1), yields

$$ \Vert \boldsymbol{\tau }_w^n\Vert _2 + \Vert \boldsymbol{\tau }_p^{n-\frac{1}{2}}\Vert _2 + |\tau _\psi ^{n-\frac{1}{2}}| \le C_\tau \,k^2, $$

for $t_n \le T$, where $C_\tau > 0$ depends on solution regularity and $\sup _{\mathcal {D}}\Vert \textbf{g}\Vert _2$. The constant is finite because the solution remains in the strict interior of $\mathcal {D}$.

### Global error

Define the grid errors $\textbf{e}_w^n \triangleq \textbf{w}^n - \textbf{w}(t_n)$, $\textbf{e}_p^{n+\frac{1}{2}} \triangleq \textbf{p}^{n+\frac{1}{2}} - \textbf{p}(t_{n+\frac{1}{2}})$, and $e_\psi ^{n+\frac{1}{2}} \triangleq \psi ^{n+\frac{1}{2}} - \psi (t_{n+\frac{1}{2}})$. The bounds $C_p$ and $C_\psi$ from (B5) guarantee that the numerical trajectory stays in a compact set $\mathcal {D}_k \subset \mathcal {D}$ for $t_n \le T$. Since $\mathcal {D}_k \subset \mathcal {D}$, the bound $G_0$ and Lipschitz constant $\mathcal {C}_g$ of $\textbf{g}$ introduced in Appendix A remain valid on $\mathcal {D}_k$. Subtracting the truncation-error relations from (B4) gives the error equations, whose nonlinear residuals satisfy

$$ \begin{aligned}&\bigl \Vert \textbf{g}(\textbf{w}^n)\,\mu _{ t+}\psi ^{n-\frac{1}{2}} \\&\quad - \textbf{g}(\textbf{w}(t_n))\, \mu _{ t+}\psi (t_{n-\frac{1}{2}})\bigr \Vert _2 \\&\qquad \le C_\psi \,\mathcal {C}_g\,\Vert \textbf{e}_w^n\Vert _2 + G_0\,|\mu _{ t+}e_\psi ^{n-\frac{1}{2}}|, \end{aligned} $$

$$ \begin{aligned}&\bigl |\textbf{g}(\textbf{w}^n)^\intercal \mu _{ t+}\textbf{p}^{n-\frac{1}{2}} \\&\quad - \textbf{g}(\textbf{w}(t_n))^\intercal \mu _{ t+}\textbf{p}(t_{n-\frac{1}{2}})\bigr | \\&\qquad \le C_p\,\mathcal {C}_g\,\Vert \textbf{e}_w^n\Vert _2 + G_0\,\Vert \mu _{ t+}\textbf{e}_p^{n-\frac{1}{2}}\Vert _2. \end{aligned} $$

Introducing the scalar error measure

$$ E^n \triangleq \Vert \textbf{e}_w^n\Vert _2 + \Vert \textbf{e}_p^{n-\frac{1}{2}}\Vert _2 + |e_\psi ^{n-\frac{1}{2}}|, $$

using $\Vert \mu _{ t+}\textbf{e}_p^{n-\frac{1}{2}}\Vert _2 \le \frac{1}{2}( \Vert \textbf{e}_p^{n+\frac{1}{2}}\Vert _2 + \Vert \textbf{e}_p^{n-\frac{1}{2}}\Vert _2)$ (analogously for $e_\psi$), one obtains the recursion

$$ E^{n+1} \le (1 + k\,C_L)\,E^n + C_\tau \,k^3, $$

where $C_L > 0$ depends on $(1\!-\!\sigma )\,\lambda _{\max }(\textbf{K})$, $\mathcal {C}_g$, $G_0$, $C_p$, and $C_\psi$. The discrete Grönwall lemma then gives, for $T = Nk$,

$$ \begin{aligned} \max _{0 \le n \le N} E^n&\le \bigl (\textrm{e}^{C_L T} C_{\textrm{in}} \\&\quad + \tfrac{C_\tau }{C_L} (\textrm{e}^{C_L T} - 1)\bigr )\,k^2 \\&\triangleq C(T)\,k^2, \end{aligned} $$

where $C_{\textrm{in}}$ accounts for the second-order initialisation error. Thus, under (26) and Lipschitz continuity of $\textbf{g}$ on $\mathcal {D}_k$, scheme (B4) is second-order convergent on any finite interval.

### Lipschitz continuity of $\textbf{g}$ and $\varepsilon$-dependence

A sufficient condition for Lipschitz continuity of $\textbf{g}$ is boundedness of its Jacobian. From $\textbf{g}= \nabla \Phi / \sqrt{2\Phi + \varepsilon }$ one has

B6 $$\begin{aligned} \textrm{J}\textbf{g}= \frac{\textrm{Hess}\,\Phi }{(2\Phi +\varepsilon )^{1/2}} - \frac{\nabla \Phi \,\nabla \Phi ^\intercal }{(2\Phi +\varepsilon )^{3/2}}. \end{aligned}$$

Expression (B6) is now evaluated at the equilibrium $(u,v) = (0,0)$ for three choices of $\Phi$.

***Case 1: ***$\Phi = V$ ***(NS).*** Since $\nabla V(0,0) = \textbf{0}$ and $V(0,0) = 0$, one has $\textbf{g}(0,0) = \textbf{0}$. The second term in (B6) vanishes and $2V(0,0)+\varepsilon = \varepsilon$; using the linearisation $V \approx u^2/2 + \alpha \, v^2/2$ gives

$$ \Vert \textrm{J}\textbf{g}(0,0)\Vert = \frac{\max \{1,\alpha \}}{\sqrt{\varepsilon }}. $$

The Lipschitz constant of $\textbf{g}$ therefore grows without bound as $\varepsilon \rightarrow 0^+$.

***Case 2:*** $\Phi = R_\textrm{A}$ ***(S, Form A).*** Similarly $\nabla R_\textrm{A}(0,0) = \textbf{0}$ and $R_\textrm{A}(0,0) = 0$. The expansion $R_\textrm{A} \approx (\alpha -1)\,v^2/2$ yields $\textrm{Hess}\,R_\textrm{A}(0,0) = (\alpha \!-\!1)\,\textrm{diag}(0,1)$, and the same mechanism gives

$$ \Vert \textrm{J}\textbf{g}(0,0)\Vert = \frac{\alpha - 1}{\sqrt{\varepsilon }}, $$

so $\textrm{J}\textbf{g}$ again diverges as $\varepsilon \rightarrow 0^+$.

***Case 3:*** $\Phi = R_\textrm{B}$ ***(S, Form B).*** The shifted residual (16b) satisfies $R_\textrm{B}(0,0) = (\alpha \!-\!1)/2 > 0$, so that $2R_\textrm{B}(0,0) + \varepsilon = \alpha - 1 + \varepsilon$ is bounded away from zero independently of $\varepsilon$. Moreover $\nabla R_\textrm{B}(0,0) = \textbf{0}$ and the first non-constant correction is cubic, giving $\nabla R_\textrm{B} = O(\Vert \textbf{w}\Vert ^2)$ and $\textrm{Hess}(R_\textrm{B}) = O(\Vert \textbf{w}\Vert )$. Substituting into (B6), both terms vanish at the origin:

$$ \Vert \textrm{J}\textbf{g}(u,v)\Vert \le C\,\left( \sqrt{u^2+v^2}\,\right) ^{\!4}, $$

for a constant $C > 0$ independent of $\varepsilon$ in a neighbourhood of (0, 0). Consequently, $\textrm{J}\textbf{g}$ remains bounded as $\varepsilon \rightarrow 0^+$—a direct consequence of the fact that $R_\textrm{B}$ does not vanish at the equilibrium (see Fig. 1).

## Appendix C Summary of continuous models

Table 5 presents the quadratised potential gradients derived from the potentials discussed in Sects. 5.2, 5.3, and 5.4. Table 6 presents the spatially discretised components of the potentials. Table 7 reports discrete versions of the quadratised potential gradients from Table 5.

**Table 5:** Discrete versions of the string potential components from Sects. 5.2, 5.3 and 5.4

	Discrete string potential components
$\mathrm {G_A}$	$$\displaystyle \begin{aligned} Q_{\mathrm {G_A}}&= \tfrac{T_0h}{2} \left( \boldsymbol{\zeta }^\intercal \boldsymbol{\zeta }+ \boldsymbol{\xi }^\intercal \boldsymbol{\xi }\right) + \tfrac{EIh}{2} \boldsymbol{\chi }^\intercal \boldsymbol{\chi }\\ R_{\mathrm {G_A}}&= h\tfrac{EA-T_0}{2}\sum _{l=1}^{N-1}\left( \sqrt{\left( 1+\xi _l \right) ^2 + \zeta _l^2} -1\right) ^2 \end{aligned}$$
$\mathrm {G_B}$	$$\displaystyle \begin{aligned} Q_{\mathrm {G_B}}&= \tfrac{T_0h}{2} \boldsymbol{\zeta }^\intercal \boldsymbol{\zeta }+ \tfrac{EAh}{2}\boldsymbol{\xi }^\intercal \boldsymbol{\xi }+ \tfrac{EIh}{2} \boldsymbol{\chi }^\intercal \boldsymbol{\chi }\\ R_{\mathrm {G_B}}&= h(EA - T_0)\sum _{l=1}^{N-1} \left( \frac{\zeta _l^2}{2} + \frac{3}{2} + \xi _l - \sqrt{\left( 1+\xi _l \right) ^2 + \zeta _l^2} \right) \end{aligned}$$
$\textrm{C}$	$$\displaystyle \begin{aligned} Q_\textrm{C}&= \tfrac{T_0h}{2} \boldsymbol{\zeta }^\intercal \boldsymbol{\zeta }+ \tfrac{EIh}{2} \boldsymbol{\chi }^\intercal \boldsymbol{\chi }\\ R_\textrm{C}&= h\tfrac{EA-T_0}{8}\left( \boldsymbol{\zeta }^2\right) ^\intercal \boldsymbol{\zeta }^2 \end{aligned}$$
$\textrm{K}$	$$\displaystyle \begin{aligned} Q_\textrm{K}&= \tfrac{T_0h}{2} \boldsymbol{\zeta }^\intercal \boldsymbol{\zeta }+ \tfrac{EIh}{2} \boldsymbol{\chi }^\intercal \boldsymbol{\chi }\\ R_\textrm{K}&= \tfrac{EAh^2}{8\,L} \left( \boldsymbol{\zeta }^\intercal \boldsymbol{\zeta }\right) ^2, \end{aligned}$$

**Table 6:** Discrete versions of the potential gradients in Table 5

	Discrete quadratised potential gradients
$\textrm{G}$	$$\displaystyle \textbf{g}_\textrm{G} =- \begin{bmatrix} T_0\textbf{D}^2\textbf{u}- EI \textbf{D}^4\textbf{u}+ (EA-T_0) \textbf{D}^+\left[ \tfrac{\boldsymbol{\zeta }\left( \sqrt{(1+\boldsymbol{\xi })^2 + \boldsymbol{\zeta }^2} - 1\right) }{\sqrt{(1+\boldsymbol{\xi })^2 + \boldsymbol{\zeta }^2}}\right] \\ T_0\textbf{D}^2\textbf{v}+ (EA - T_0)\textbf{D}^+\left[ \tfrac{(1+\boldsymbol{\xi })\left( \sqrt{(1+\boldsymbol{\xi })^2 + \boldsymbol{\zeta }^2} - 1\right) }{\sqrt{(1+\boldsymbol{\xi })^2 + \boldsymbol{\zeta }^2}}\right] \end{bmatrix} \left( 2V_\textrm{G}+\varepsilon \right) ^{-\tfrac{1}{2}}$$
$\mathrm {G_A}$	$$\displaystyle {{\tilde{\textbf{g}}}}_\mathrm {G_A}=-(EA - T_0) \begin{bmatrix} \textbf{D}^+\left[ \tfrac{\boldsymbol{\zeta }\left( \sqrt{(1+\boldsymbol{\xi })^2 + \boldsymbol{\zeta }^2} - 1\right) }{\sqrt{(1+\boldsymbol{\xi })^2 + \boldsymbol{\zeta }^2}}\right] \\ \textbf{D}^+\left[ \tfrac{(1+\boldsymbol{\xi })\left( \sqrt{(1+\boldsymbol{\xi })^2 + \boldsymbol{\zeta }^2} - 1\right) }{\sqrt{(1+\boldsymbol{\xi })^2 + \boldsymbol{\zeta }^2}}\right] \end{bmatrix}\left( 2\textrm{R}_\mathrm {G_A}+\varepsilon \right) ^{-\tfrac{1}{2}}$$
$\mathrm {G_B}$	$$\displaystyle {{\tilde{\textbf{g}}}}_\mathrm {G_B}=- (EA - T_0) \begin{bmatrix} \textbf{D}^+\left[ \boldsymbol{\zeta }- \tfrac{\boldsymbol{\zeta }}{\sqrt{(1+\boldsymbol{\xi })^2 + \boldsymbol{\zeta }^2}}\right] \\ \textbf{D}^+\left[ 1 -\tfrac{\boldsymbol{\xi }+1 }{\sqrt{(1+\boldsymbol{\xi })^2 + \boldsymbol{\zeta }^2}}\right] \end{bmatrix}\left( 2\textrm{R}_\mathrm {G_B}+\varepsilon \right) ^{-\tfrac{1}{2}}$$
C	$\displaystyle \textbf{g}_\textrm{C}=- \left[ T_0\textbf{D}^2\textbf{u}- EI\textbf{D}^4\textbf{u}+\textbf{D}^+\left( \tfrac{EA-T_0}{2}\boldsymbol{\zeta }^3 \right) \right] \left( 2V_\textrm{C}+\varepsilon \right) ^{-\tfrac{1}{2}}$
C-S	$\displaystyle {\tilde{\textbf{g}}}_\textrm{C}=- \textbf{D}^+\left( \tfrac{EA-T_0}{2}\boldsymbol{\zeta }^3 \right) \left( 2 R_\textrm{C}+\varepsilon \right) ^{-\tfrac{1}{2}}$
K	$\displaystyle \textbf{g}_\textrm{K}=- \left[ T_0\textbf{D}^2\textbf{u}- EI\textbf{D}^4\textbf{u}+ \tfrac{EAh}{2L} \textbf{D}^+\boldsymbol{\zeta }\left( \boldsymbol{\zeta }^\intercal \boldsymbol{\zeta }\right) \right] \left( 2 V_\textrm{K}+\varepsilon \right) ^{-\tfrac{1}{2}}$
K-S	$\displaystyle {\tilde{\textbf{g}}}_\textrm{K}=- \tfrac{EAh}{2L}\textbf{D}^+\boldsymbol{\zeta }\left( \boldsymbol{\zeta }^\intercal \boldsymbol{\zeta }\right) \left( 2 R_\textrm{K}+\varepsilon \right) ^{-\tfrac{1}{2}}$

## Code Availability

The code used for this study is openly available on Zenodo at https://doi.org/10.5281/zenodo.20413144.

## References

[1] Morse, P., Ingard, U.: Theoretical Acoustics. Princeton University Press, Princeton, NJ, USA (1968)

[2] Kurmyshev, E.V.: Transverse and longitudinal mode coupling in a free vibrating soft string. Phys. Lett. A **310**(2–3), 148–160 (2003). 10.1016/S0375-9601(03)00264-0

[3] Kirchhoff, G.: Vorlesungen über Mechanic. B.G. Teubner, Leipzig, Germany (1883)

[4] Carrier, G.F.: On the non-linear vibration problem of the elastic string. Q. Appl. Math. **3**, 157–165 (1945). 10.1090/QAM/12351

[5] Anand, G.V.: Nonlinear resonance in stretched strings with viscous damping. J. Acoust. Soc. Am. **40**(6), 1517–1528 (1966). 10.1121/1.1910257

[6] Murthy, G.S.S., Ramakrishna, B.S.: Nonlinear character of resonance in stretched strings. J. Acoust. Soc. Am. **38**(3), 461–471 (1965). 10.1121/1.1909715

[7] Bilbao, S.: Numerical Sound Synthesis. John Wiley & Sons Ltd, Chichester, UK (2009)

[8] Gough, C.: The nonlinear free vibration of a damped elastic string. J. Acoust. Soc. Am. **75**(6), 1770–1776 (1984). 10.1121/1.390977

[9] Legge, K.A., Fletcher, N.H.: Nonlinear generation of missing modes on a vibrating string. J. Acoust. Soc. Am. **76**(1), 5–12 (1984). 10.1121/1.391007

[10] Narasimha, R.: Non-linear vibration of an elastic string. J. Sound Vib. **8**(1) (1968) 10.1016/0022-460X(68)90200-9

[11] Chabassier, J., Joly, P.: Energy preserving schemes for nonlinear Hamiltonian systems of wave equations: application to the vibrating piano string. Comput. Methods Appl. Mech. Eng. **199**(45), 2779–2795 (2010). 10.1016/j.cma.2010.04.013

[12] Conklin, H.A.: Generation of partials due to nonlinear mixing in a stringed instrument. J. Acoust. Soc. Am. **105**(1), 536–545 (1999). 10.1121/1.424589

[13] Bilbao, S.: Conservative numerical methods for nonlinear strings. J. Acoust. Soc. Am. **118**(5), 3316–3327 (2005). 10.1121/1.2046787

[14] Anand, G.V.: Large-amplitude damped free vibration of a stretched string. J. Acoust. Soc. Am. **45**(5), 1089–1096 (1969). 10.1121/1.1911578

[15] Bilbao, S., Ducceschi, M.: Models of musical string vibration. Acoust. Sci. Technol. **44**(3), 194–209 (2023). 10.1250/ast.44.194

[16] Betsch, P., Steinmann, P.: Conservation properties of a time FE method-part II: Time-stepping schemes for non-linear elastodynamics. Int. J. Numer. Meth. Eng. **50**(8), 1931–1955 (2001). 10.1002/nme.103

[17] Bilbao, S.: Modal type synthesis techniques for nonlinear strings with an energy conservation property. In: Proceedings of the International Conference on Digital Audio Effects (DAFx), pp. 119–124. , Naples, Italy (2004)

[18] Chabassier, J., Chaigne, A., Joly, P.: Modeling and simulation of a grand piano. J. Acoust. Soc. Am. **134**(1), 648–665 (2013). 10.1121/1.4809649 23862839 10.1121/1.4809649

[19] Chabassier, J.: Modélisation et simulation numérique d’un piano par modèles physiques, Ecole Polytechnique (2012). (PhD thesis)

[20] Chatziioannou, V., Schmutzhard, S., Bilbao, S.: On iterative solutions for numerical collision models. In: Proceedings of the International Conference on Digital Audio Effects (DAFx), pp. 72–79. , Edinburgh, UK (2017)

[21] Fontana, F., Bozzo, E.: Newton-Raphson solution of nonlinear delay-free loop filter networks. IEEE/ACM Trans. Audio Speech Lang. Process. **27**(10), 1590–1600 (2019). 10.1109/TASLP.2019.2924842

[22] Lopes, N., Hélie, T., Falaize, A.: Explicit second-order accurate method for the passive guaranteed simulation of port-Hamiltonian systems. In: Proceedings of the IFAC Workshop on Lagrangian and Hamiltonian Methods for Nonlinear Control (IFAC), Lyon, France, pp. 223–228 (2015). 10.1016/j.ifacol.2015.10.243

[23] Roze, D., Raibaud, M., Geoffroy, T.: Passive-guaranteed modeling and simulation of a finite element nonlinear string model. In: Proceedings of the IFAC Workshop on Lagrangian and Hamiltonian Methods for Non Linear Control (LHMNC), Besançon, France, pp. 226–231 (2024). 10.1016/j.ifacol.2024.08.285

[24] Yang, X.: Linear, first and second-order, unconditionally energy stable numerical schemes for the phase field model of homopolymer blends. J. Comput. Phys. **327**, 294–316 (2016). 10.1016/j.jcp.2016.09.029

[25] Zhao, J., Wang, Q., Yang, X.: Numerical approximations for a phase field dendritic crystal growth model based on the invariant energy quadratization approach. Int. J. Numer. Meth. Eng. **110**(3), 279–300 (2017). 10.1002/nme.5372

[26] Yang, X., Ju, L.: Linear and unconditionally energy stable schemes for the binary fluid-surfactant phase field model. Comput. Methods Appl. Mech. Eng. **318**, 1005–1029 (2017). 10.1016/j.cma.2017.02.011

[27] Shen, J., Xu, J., Yang, J.: The scalar auxiliary variable (SAV) approach for gradient flows. J. Comput. Phys. **353**, 407–416 (2018). 10.1016/j.jcp.2017.10.021

[28] Shen, J., Xu, J.: Convergence and error analysis for the scalar auxiliary variable (sav) schemes to gradient flows. SIAM J. Numer. Anal. **56**(5), 2895–2912 (2018). 10.1137/17M1159968

[29] Shen, J., Xu, J., Yang, J.: A new class of efficient and robust energy stable schemes for gradient flows. SIAM Rev. **61**(3), 474–506 (2019). 10.1137/17M1150153

[30] Bilbao, S., Ducceschi, M.: Fast explicit algorithms for Hamiltonian numerical integration. In: Proceedings of 2020 the European Nonlinear Dynamics Conference, , Lyon, France (2022)

[31] Bilbao, S., Ducceschi, M., Zama, F.: Explicit exactly energy-conserving methods for Hamiltonian systems. J. Comput. Phys. **472**, 111697 (2023). 10.1016/j.jcp.2022.111697

[32] Sherman, J., Morrison, W.J.: Adjustment of an inverse matrix corresponding to a change in one element of a given matrix. Ann. Math. Stat. **21**, 124–127 (1950). 10.1214/aoms/1177729893

[33] Russo, R., Bilbao, S., Duccheschi, M.: Scalar auxiliary variable techniques for nonlinear transverse string vibration. In: Proceedings of the IFAC Workshop on Lagrangian and Hamiltonian Methods for Non Linear Control (IFAC), Besançon, France, pp. 160–165 (2024). 10.1016/j.ifacol.2024.08.274

[34] Russo, R.: Non-iterative numerical simulation techniques for nonlinear string vibration in musical acoustics, Bologna, Italy (2025). https://doi.org/10.48676/unibo/amsdottorato/12127 . (Phd dissertation)

[35] Russo, R., Webb, C.J., Ducceschi, M., Bilbao, S.: Convergence analysis and relaxation techniques for modal scalar auxiliary variable methods applied to nonlinear transverse string vibration. Proc. Meetings Acoust. **56**(1), 035007 (2025). 10.1121/2.0002073

[36] Ducceschi, M., Bilbao, S.: Real-time simulation of the struck piano string with geometrically exact nonlinearity via novel quadratic Hamiltonian method. In: Proceedings of the 2020 European Nonlinear Dynamics Conference, , Lyon, France (2022)

[37] Castera, Guillaume, Chabassier, Juliette: Linearly implicit time integration scheme of lagrangian systems via quadratization of a nonlinear kinetic energy. application to a rotating flexible piano hammer shank. ESAIM: M2AN 58(5), 1881–1905 (2024) 10.1051/m2an/2024049

[38] Castera, G.: Modélisation, analyse numérique et simulation de la propagation des ondes longitudinales dans le piano, Université de Pau et des Pays de l’Adour (2023). (Application à l&apos;étude du toucher instrumental. PhD thesis)

[39] Brugnoli, A., Matignon, D., Morlier, J.: A linearly-implicit energy-momentum preserving scheme for geometrically nonlinear mechanics based on non-canonical hamiltonian formulations. Nonlinear Dyn. **113**(20), 27539–27566 (2025). 10.1007/s11071-025-11601-6

[40] Ducceschi, M., Bilbao, S.: Non-iterative solvers for nonlinear problems: The case of collisions. In: Proceedings of the Internatinal Conference on Digital Audio Effects (DAFx), pp. 17–24. , Birmingham, UK (2019)

[41] Van Walstijn, M., Chatziioannou, V., Bhanuprakash, A.: Implicit and explicit schemes for energy-stable simulation of string vibrations with collisions: refinement, analysis, and comparison. Journal of Sound and Vibration 569, 117968 (2024) 10.1016/j.jsv.2023.117968

[42] Risse, T., Helie, T., Bilbao, S.: Power-balanced drift regulation for scalar auxiliary variable methods: application to real-time simulation of nonlinear string vibrations. In: Proceedings of the International Conference on Digital Audio Effects (DAFx), pp. 126–133. , Ancona, Italy (2025)

[43] Zama, F., Ducceschi, M., Bilbao, S.: Role of shift constant in energy shifted SAV for Hamiltonian systems. J. Phys: Conf. Ser. **2701**(1), 012089 (2024). 10.1088/1742-6596/2701/1/012089

[44] Courant, R., Friedrichs, K., Lewy, H.: On the partial difference equations of mathematical physics. IBM J. Res. Dev. **11**(2), 215–234 (1967)

[45] Ducceschi, M., Bilbao, S.: Linear stiff string vibrations in musical acoustics: Assessment and comparison of models. J. Acoust. Soc. Am. **140**(4), 2445–2454 (2016). 10.1121/1.4962553 27794349 10.1121/1.4962553

[46] Antman, S.S.: Nonlinear Problems of Elasticity, 2nd edn. Applied Mathematical Sciences, vol. 107. Springer, New York, NY, USA (2005). 10.1007/0-387-27649-1

[47] Ducceschi, M., Bilbao, S.: Simulation of the geometrically exact nonlinear string via energy quadratisation. J. Sound Vib. **534**, 117021 (2022). 10.1016/j.jsv.2022.117021

